# Extensive Genetic Diversity and Widespread Azole Resistance in Greenhouse Populations of Aspergillus fumigatus in Yunnan, China

**DOI:** 10.1128/mSphere.00066-21

**Published:** 2021-02-10

**Authors:** Duanyong Zhou, Greg A. Korfanty, Meizi Mo, Ruirui Wang, Xiao Li, Haixia Li, Shuoshuo Li, Jin-Yan Wu, Ke-Qin Zhang, Ying Zhang, Jianping Xu

**Affiliations:** a State Key Laboratory for Conservation and Utilization of Bio-Resources in Yunnan, Yunnan University, Kunming, People’s Republic of China; b College of Life Science, Yunnan University, Kunming, People’s Republic of China; c School of Biology and Chemistry, Xingyi Normal University for Nationalities, Xingyi, People’s Republic of China; d Department of Biology, McMaster University, Hamilton, Ontario, Canada; e Public Research Laboratory, Hainan Medical University, Haikou, People’s Republic of China; f Key Laboratory for Southwest Microbial Diversity of the Ministry of Education, Yunnan University, Kunming, People’s Republic of China; University of Georgia

**Keywords:** short tandem repeats, genetic clustering, azole susceptibility, *cyp51A*, fungicide residue

## Abstract

Aspergillus fumigatus is the main cause of invasive aspergillosis (IA) with a high annual global incidence and mortality rate. Recent studies have indicated an increasing prevalence of azole-resistant A. fumigatus (ARAF) strains, with agricultural use of azole fungicides as a potential contributor. China has an extensive agricultural production system and uses a wide array of fungicides for crop production, including in modern growth facilities such as greenhouses. Soils in greenhouses are among the most intensively cultivated. However, little is known about the occurrence and distribution of ARAF in greenhouse soils. Here, we investigated genetic variation and triazole drug susceptibility in A. fumigatus from greenhouses around metropolitan Kunming in Yunnan, southwest China. Abundant allelic and genotypic variations were found among 233 A. fumigatus strains isolated from nine greenhouses in this region. Significantly, ∼80% of the strains were resistant to at least one medical triazole drug, with >30% showing cross-resistance to both itraconazole and voriconazole. Several previously reported mutations associated with triazole resistance in the triazole target gene *cyp51A* were also found in our strains, with a strong positive correlation between the frequency of mutations at the *cyp51A* promoter and that of voriconazole resistance. Phylogenetic analyses of *cyp51A* gene sequences showed evidence for multiple independent origins of azole-resistant genotypes of A. fumigatus in these greenhouses. Evidence for multiple origins of azole resistance and the widespread distributions of genetically very diverse triazole-resistant strains of A. fumigatus in greenhouses calls for significant attention from public health agencies.

**IMPORTANCE** The origin and prevalence of azole-resistant Aspergillus fumigatus have been attracting increasing attention from biologists, clinicians, and public health agencies. Current evidence suggests agricultural fungicide use as a major cause. In southwest China, greenhouses are used to produce large amounts of fruits, flowers, and vegetables for consumers throughout China as well as those in other countries, primarily in southeast Asia. Here, we found a very high frequency (∼80%) of triazole-resistant A. fumigatus in our sample, the highest reported so far, with a significant proportion of these strains resistant to both tested agricultural fungicides and medical triazole drugs. In addition, we found novel allelic and genotypic diversities and evidence for multiple independent origins of azole-resistant genotypes of A. fumigatus in greenhouse populations in this region. Our study calls for a systematic evaluation of the effects of azole fungicide usage in greenhouses on human health.

## INTRODUCTION

*Aspergillus* is a large fungal genus comprising more than 300 different species distributed in a broad range of ecological niches. *Aspergillus* species can release large amounts of airborne asexual spores, the conidia, into the environment. Thus, inhalation of *Aspergillus* conidia by humans is a common event. While such spores typically have no or very limited effects on healthy people, for immunocompromised individuals, such exposures can result in significant infections. The most severe form of *Aspergillus* infection is invasive aspergillosis (IA). IA is commonly caused by Aspergillus fumigatus and has a global annual incidence of approximately 10% and a mortality rate as high as 90% (1–3), even when properly diagnosed and treated (4, 5). Successful treatments of IA have relied almost exclusively on triazole antifungals, such as itraconazole (ITR), posaconazole (POS), and voriconazole (VOR) (6, 7). However, prolonged use of triazoles increases the likelihood of the development of drug resistance (8). Unfortunately, since the first report of ITR-resistant A. fumigatus in 1997 (9), azole-resistant A. fumigatus (ARAF) has been widely identified both in IA patients and in environmental isolates from many parts of the world (10–13). The recent COVID-19 pandemic has further exacerbated the problem, with increasing reports of COVID-19-associated azole-resistant pulmonary aspergillosis (CAPA) affecting patients with severe pulmonary abnormalities and causing prolonged stays in intensive care units (14, 15).

Due to their clinical importance, significant efforts have been put into investigating the reasons for the origin of ARAF strains. Consequently, our understanding of the origin and distributions of ARAF has improved markedly in the last 2 decades. At the molecular level, a number of point mutations in the *cyp51A* gene causing amino acid substitutions in lanosterol 14-α-demethylase, the target of triazole drugs, are known to be associated with reduced triazole susceptibility. Specifically, the amino acids at positions G54, Y121, G138, P216, F219, M220, A284, Y431, G432, G434, and G448 represent the known major azole resistance mutations in *Aspergillus* (16–22). Second, transcriptional upregulation of *cyp51A* by changes in tandem repeats TR_34_, TR_46_, and TR_53_ in the promoter region is frequently observed among azole-resistant strains. Some of the tandem repeats in the promoter region co-occur with specific single nucleotide polymorphisms (SNPs) in the coding sequence to confer azole resistance. Specifically, two such promoter expansion and nonsynonymous substitution combinations have been widely observed, TR_34_/L98H and TR_46_/Y121F/T289A (20, 23–26). In addition, overexpression of multidrug efflux pumps has been hypothesized as being responsible for the acquisition of azole resistance in *Aspergillus*. Such efflux pumps can reduce the amount of triazole drugs within cells and maintain regular cellular activities (27). However, despite the reported associations of many mutations at the *cyp51A* locus in azole-resistant strains of A. fumigatus, few have been validated, and the detailed molecular mechanisms for azole resistance remain incompletely understood.

Understanding the genetic diversity and population history of pathogenic fungi can help us determine the origins and spread of antifungal resistance. Such knowledge could help us develop better prevention and control measures against the origins and transmissions of drug-resistant infections (28). Indeed, over the last few decades, environmental and clinical A. fumigatus strains from different geographic areas have been genotyped with multiple molecular methods, including multilocus sequence typing (MLST), short tandem repeats (STRs) or microsatellite markers, randomly amplified polymorphic DNA (RAPD) typing, restriction fragment length polymorphisms (RFLP) detected through Southern hybridization, PCR-RFLP, amplified fragment length polymorphisms (AFLP), and whole-genome sequencing (29–34). These studies have revealed variable results, from no genetic structure to significant genetic structuring and multiple distinct clusters, depending on the markers used and the population samples analyzed (35). Recently, simple and low-cost genotyping methods such as ARMS-PCR using tetra-primers and cell surface protein typing (CSP) were developed to detect and genotype ARAFs (36, 37). Among these markers, due to their high reproducibility and high discriminating power, a panel of nine STRs has emerged as the markers of choice for genotyping A. fumigatus. Indeed, these nine markers have been used to analyze local, regional, and global samples of A. fumigatus (1, 5, 38). In a recent study, two well-supported phylogenetic clades were identified in the global A. fumigatus populations (25, 31, 39–41). Studies based on both high-resolution whole-genome sequencing of dozens of isolates and STR genotyping of a global collection of 4,049 isolates found that isolates harboring the TR_34_/L98H or the TR_46_/Y121F/T289A mutations were not evenly distributed between the two phylogenetic clades. These results suggest that genetic backgrounds may be related to the propensity of certain strains to develop resistance to multiple azole drugs, including the TR_34_/L98H mutation. Alternatively, such mutations could cause selective sweeps and change the genetic structure of natural populations of A. fumigatus, leading to their biased distributions between clades (5, 25, 42).

So far, both clinical and environmental samples of ARAF have been found from Europe, America, Africa, New Zealand, the Middle East, and Asia (27, 43–52). Traditionally, the emergence of azole resistance in A. fumigatus populations was believed due to long-term clinical azole therapy. Since ARAF can also be isolated from patients who had never received azole therapy (53), environmental origins of ARAF have been increasingly suspected for these patients. Indeed, azole fungicides have molecular targets identical/similar to those of clinical azole drugs against A. fumigatus (27), and a large number of agricultural azole fungicides have been used in many parts of the world. In China, azole fungicides triadimefon (TRI), tebuconazole (TEB), difenoconazole (DIF), propiconazole (PRO), hexaconazole (HEX), and flusilazole (FLU) are the top six most commonly used fungicides (54). Based on samples isolated from 12 provinces in China, 2.5% and 1.4% of clinical and environmental A. fumigatus strains, respectively, were identified as resistant to azoles (55). Though the genetic features and drug-resistant mutations were unknown, a study analyzing A. fumigatus in Zhejiang Province, China, found ARAF in soil samples from greenhouses (27). Furthermore, TR_34_/L98H and TR_34_/L98H/S297T/F495I were found to be the most common resistance mechanisms in China. However, strains with these two mutations were phylogenetically distinct from those strains with the same mutations in the Netherlands and Denmark as indicated by STR typing, consistent with their independent origins (55). These results suggest that the use of azole fungicides has likely selected for the development of azole resistance among fungal species in soils. The increasing antifungal resistance in agricultural fields can cause significant problems not only for agriculture but also for human health, especially in the case of opportunistic human fungal pathogens with a primary ecological niche in soil, including agricultural soil. Thus, it is very important to investigate the prevalence and molecular mechanisms of azole resistance in order to better understand the origin and evolution of azole resistance and to better respond to the continued emergence of azole-resistant aspergillosis.

The objective of this study was to analyze the fine-scale population structure of A. fumigatus samples from greenhouses in southwestern China. At present, almost nothing is known about the prevalence of azole resistance in A. fumigatus from southwest China. Due to its relatively mild climate throughout the year, Yunnan Province in southwestern China produces large amounts of fruits, flowers, and vegetables for consumers throughout China. Some of those horticultural and agricultural products are also exported to other countries. The sustained high agricultural productivity in this region has benefited from the presence of a large number of greenhouses, especially around Kunming, the capital city of Yunnan Province. In this study, we isolated and analyzed A. fumigatus from nine greenhouses around Kunming and compared them with each other and with those from other parts of the world in the global database. Our study identified a high prevalence of azole-resistant strains, with a significant proportion resistant to two medical triazole drugs. The DNA sequences at the azole target gene *cyp51A* were analyzed to identify potential mutations associated with the observed azole resistance.

## RESULTS

### Isolation and susceptibility of A. fumigatus isolates.

From the 900 soil samples from the nine different greenhouses, we obtained a total of 233 A. fumigatus isolates, each from a different soil sample. Analyses of the internal transcribed spacer (ITS) sequences for 60 randomly selected isolates representing all nine greenhouses confirmed that all 60 belonged to A. fumigatus. Our successful STR genotyping using the nine A. fumigatus-specific primers also confirmed the correct species identification. Furthermore, the STR genotyping assays showed that each isolate contained one allele at each of the nine STR loci, consistent with each of the 233 strains being a single pure isolate. The numbers of A. fumigatus isolates from greenhouse populations 1 to 9 (pop. 1 to pop. 9) were 28, 25, 28, 27, 24, 28, 20, 25, and 28, respectively. Azole susceptibility testing of these isolates showed that the MICs of ITR, VOR, TRI, and TEB ranged from 1 to ≥16 μg/ml (MIC_50_ = 8 μg/ml), 0.125 to ≥16 μg/ml (MIC_50_ = 1 μg/ml), 32 to >32 μg/ml, and 0.125 to ≥32 μg/ml (MIC_50_ = 4 μg/ml), respectively (see Table S1 in the supplemental material). In total, for the two clinical triazoles ITR and VOR, 78.54% (183/233), 33.91% (79/233), and 33.48% (78/233) of the A. fumigatus isolates were able to grow at drug concentrations higher than the resistance breakpoint values of ITR, VOR, and both ITR and VOR, respectively. Interestingly, the prevalence of azole-resistant strains varied widely among the greenhouses for different azoles. For example, all strains isolated from pop. 1, pop. 6, and pop. 9 were ITR resistant, while the prevalence of ITR resistance in pop. 3 was only 32.14% (9/28). The most abundant resistant A. fumigatus strains to VOR were found in pop. 9 at 92.86% (26/28), while no strain in pop. 2 was resistant to VOR. For triazole fungicides, 100% (233/233) and 28.76% (67/233) A. fumigatus isolates were able to grow on media with ≥16 μg/ml (MIC ≥ 32 μg/ml) TRI and TEB, respectively. Interestingly, similar to those for ITR and VOR but different from that for TRI, the prevalence for TEB resistance varied widely among the nine greenhouse populations, with 89.29% (25/28) of strains isolated from pop. 1 showing an MIC of ≥32 μg/ml, while no strain in pop. 4 showed such a high MIC. Our comparative analyses of the susceptibility patterns to the four azole drugs showed that most greenhouses with a high proportion of ITR resistance also had a high MIC value to TEB (MIC ≥ 4 μg/ml) (Table 1).

**TABLE 1:** Prevalence of azole resistance among A. fumigatus samples from the nine greenhouses

Population	No. of isolates	Proportion resistant (no. of resistant isolates/total no. of isolates) to:			Proportion of strains (no. of isolates within MIC value range/total no. of isolates) with an MIC to tebuconazole of:		
		ITR	VOR	Both ITR and VOR	≥32 μg/ml	4–16 μg/ml	<4 μg/ml
Pop. 1	28	100 (28/28)	85.71 (24/28)	85.71 (24/28)	89.29 (25//28)	10.71 (3/28)	00.00 (0/28)
Pop. 2	25	76 (19/25)	0 (0/25)	0 (0/25)	4.00 (1/25)	88.00 (22/25)	8.00 (2/25)
Pop. 3	28	32.14 (9/28)	3.57 (1/28)	3.57 (1/28)	7.14 (2/28)	50 (14/28)	42.86 (12/28)
Pop. 4	27	62.96 (17/27)	7.41 (2/27)	7.41 (2/27)	0.00 (0/27)	25.93 (7/27)	74.07 (20/27)
Pop. 5	24	45.83 (11/24)	25 (6/24)	20.83 (5/24)	8.33 (2/24)	37.50 (9/24)	54.17 (13/24)
Pop. 6	28	100 (28/28)	32.14 (9/28)	32.141 (9/28)	17.86 (5/28)	78.57 (22/23)	3.57 (1/28)
Pop. 7	20	95 (19/20)	15 (3/20)	15 (3/20)	10.00 (2/20)	60.00 (12/20)	30.00 (6/20)
Pop. 8	25	96 (24/25)	32 (8/25)	32 (8/25)	32.00 (8/25)	36.00 (9/25)	32.00 (8/25)
Pop. 9	28	100 (28/28)	92.86 (26/28)	92.86 (26/28)	78.57 (22/28)	17.86 (5/28)	3.57 (1/28)
Total	233	78.54 (183/233)	33.91 (79/233)	33.48 (78/233)	28.76 (67/233)	44.21 (103/233)	27.04 (63/233)

10.1128/mSphere.00066-21.4 TABLE S1 Azole-susceptibility profiles and insertional mutation in the promoter region of the *cyp51A* gene in A. fumigatus isolates. Download Table S1, DOC file, 0.3 MB. Copyright © 2021 Zhou et al. 2021 Zhou et al. https://creativecommons.org/licenses/by/4.0/ This is an open-access article distributed under the terms of the Creative Commons Attribution 4.0 International license.

### *cyp51A* gene sequencing and phylogenetic analysis.

Our sequence analyses of the azole target gene *cyp51A* showed that 14.16% (33/233) of A. fumigatus isolates had insertional mutations in the promoter region of the *cyp51A* gene, including TR_34_/L98H (*n* = 25; 10.73%), TR_34_/L98H/S297T/F495I (*n* = 3; 1.29%), TR_46_/Y121F/T289A (*n* = 3; 1.29%), and TR_53_ (*n* = 2; 0.86%) (Table S1).

Interestingly, similar to the variable frequencies of ARAF from different greenhouses, the occurrence of insertion mutations in the promoter region among different greenhouses also varied widely. Isolates of A. fumigatus in pop. 1 and pop. 9 were found to have the highest insertional mutations in the promoter region, at 39.29% each, while none was found in pop. 2.

Among the 233 A. fumigatus isolates, there were 17 single nucleotide polymorphisms (SNPs) in the sequences of the *cyp51*A gene, with two in the intron region and 15 in the exon region. Among the 15 SNPs in the exon regions, four were synonymous, including 267G→A, 540G→A, 1074A→G, and 1362T→C, with the frequencies of mutated bases at 0.0601 (14/233), 0.0472 (11/233), 0.9399 (219/233), and 0.0601 (14/233), respectively. Interestingly, three of the four mutations (267G→A, 1074A→G, and 1362T→C) always occurred together. The remaining 11 SNPs were nonsynonymous, which separated the 233 strains into nine haplotypes, among which the most frequent haplotype was F, with a frequency of 0.6867 (160/233), followed by haplotype E, at a frequency of 0.1202 (28/233) (Table 2). Both haplotypes F and E were shared among the nine greenhouses. Four of the haplotypes (B, C, D, and I) were shared by at least two greenhouses. Of the remaining three, two haplotypes, A and H, were only found in pop. 8, while haplotype G was only found in pop. 9.

**TABLE 2:** Information about the nine greenhouses and their populations of Aspergillus fumigatus, including the distributions of nonsynonymous substitutions at the *cyp51A* gene

Population	No. of total isolates	No. of genotypes	No. of isolates for each genotype^*a*^									Fungicide residues (mg/kg)^*b*^			Northern latitude	Eastern longitude	Kind of vegetable
			A	B	C	D	E	F	G	H	I	Triadimefon	Tebuconazole	Difenoconazole			
Pop. 1	28	6		1	1	7	4	14			1	0.00088 ± 0.00019 A	0.00882 ± 0.00101 A	0.10632 ± 0.00801 E	24.651	102.703	Coriander
Pop. 2	25	3		1			4	20				Not detected	0.00457 ± 0.00034 A	0.00328 ± 0.0002 A	24.646	102.694	Cucurbita
Pop. 3	28	3				1	2	25				0.19043 ± 0.00528 C	0.01894 ± 0.00137 A	0.02308 ± 0.00032 B	24.637	102.69	Pea
Pop. 4	27	3			1		3	23				0.00302 ± 0.00004 A	0.00805 ± 0.00064 A	0.06428 ± 0.00177 C	24.628	102.687	Lettuce
Pop. 5	24	3				2	4	18				0.00563 ± 0.00025 A	0.00754 ± 0.00073 A	0.05812 ± 0.00066 C	24.618	102.683	Fennel
Pop. 6	28	3				1	5	22				0.08827 ± 0.00178 B	0.34515 ± 0.0268 C	0.14293 ± 0.00552 F	24.61	102.68	Cucurbita
Pop. 7	20	3				1	1	18				0.00529 ± 0.00023 A	0.03089 ± 0.00266 A	0.01005 ± 0.00224 A	24.608	102.685	None (rotation intervals)
Pop. 8	25	6	10			4	2	7		1	1	0.0075 ± 0.00066 A	0.05673 ± 0.00488 B	0.0792 ± 0.00758 D	24.622	102.688	None (rotation intervals)
Pop. 9	28	6			1	9	3	13	1		1	0.00611 ± 0.00076 A	0.00251 ± 0.00039 A	0.00386 ± 0.00009 A	24.631	102.692	None (rotation intervals)
Total	233	9	10	2	3	25	28	160	1	1	3						

a A, ATAGATCATAT; B, ATAGCTGATAT; C, TAAAATCAAGA; D, TAAAATCATGT; E, TTAAAACATGT; F, TTAAATCATGT; G, TTAAATCGTGT; H, TTAGATCATGT; I, TTTAATCGTGT at the 11 nonsynonymous polymorphic nucleotide sites.

b Different letters indicate significant differences at *P* < 0.05 in the same column.

When translated into amino acids, the 11 nonsynonymous substitutions caused changes at 10 amino acid sites, including F46Y, L98H, Y121F, M172V, N248T/K, D255E, T289A, S297T, E427K, and F495I (Table 3). Further analysis revealed that 12 of the 233 strains had a combination of three nonsynonymous mutations, Y46F, E427K, and M172V, with two of the 12 having two additional mutations, N248T and D255E. Furthermore, three strains each had two (Y121F and T289A) or three (L98H, S297T, and F495I) mutations together. We analyzed the associations among the SNPs using Multilocus software. In this analysis, synonymous and nonsynonymous SNPs were analyzed separately, and different bases at the same SNP site were treated as different alleles. The results of our analyses were consistent with evidence of recombination/convergent mutation within the synonymous sites in our population of A. fumigatus. Specifically, although the analysis of the four synonymous SNPs rejected the null hypothesis of random recombination (rBarD = 0.537306, *P* < 0.001), there was clear evidence of phylogenetic incompatibility between two pairs of SNP sites (267G→A and 540G→A; 540G→A and 1074A→G) (PrC = 0.5, *P* = 0.085). In contrast, there was a lack of phylogenetic incompatibility between pairs of SNP sites among the 11 nonsynonymous SNPs (PrC = 1, *P* < 0.001; rBarD = 0.111135, *P* < 0.001), indicating no evidence of recombination or convergent mutation at these nonsynonymous SNP sites. The lack of recombination allowed us to reconstruct the ancestral nonsynonymous SNPs across the phylogeny constructed by the synonymous SNPs using RASP (see Fig. S1). Our analyses showed that isolates harboring nonsynonymous mutations were broadly distributed across the phylogeny, consistent with multiple origins of the azole-resistant genotypes. The high ratio of nonsynonymous to synonymous evolutionary changes (*dN/dS* ratio) at the *cyp51A* gene (ω = 2.0004 [0.9062/0.4530]) also suggested that positive selection was likely a major force driving the origin and maintenance of azole-resistant *cyp51A* mutations.

**TABLE 3:** Distribution of amino acid substitutions within the *cyp51A* gene among the 233 A. fumigatus isolates from nine greenhouses

Mutation site	Mutation type	No. of strains with the mutation in each greenhouse										Frequency (for 233 isolates)
		Pop. 1	Pop. 2	Pop. 3	Pop. 4	Pop. 5	Pop. 6	Pop. 7	Pop. 8	Pop. 9	Total	
46	F→Y	1	1	0	0	0	0	0	10	0	12	0.0515
98	L→H	8	0	1	1	2	1	1	4	10	28	0.1202
121	Y→F	1	0	0	0	0	0	0	1	1	3	0.0129
172	M→V	1	1	0	0	0	0	0	10	0	12	0.0515
248	N→K	4	4	2	3	4	5	1	2	3	28	0.1202
248	N→T	1	1	0	0	0	0	0	0	0	2	0.0086
255	D→E	1	1	0	0	0	0	0	0	0	2	0.0086
289	T→A	1	0	0	0	0	0	0	1	2	4	0.0172
297	S→T	1	0	0	1	0	0	0	0	1	3	0.0129
427	E→K	1	1	0	0	0	0	0	10	0	12	0.0515
495	F→I	1	0	0	1	0	0	0	0	1	3	0.0129

10.1128/mSphere.00066-21.1 FIG S1 Ancestral reconstruction of nonsynonymous *cyp51A* sites for the 233 greenhouse A. fumigatus strains in this study. Letters “A” to “I” represent genotypes of nonsynonymous *cyp51A* sites: A, ATAGATCATAT; B, ATAGCTGATAT; C, TAAAATCAAGA; D, TAAAATCATGT; E, TTAAAACATGT; F, TTAAATCATGT; G, TTAAATCGTGT; H, TTAGATCATGT; I, TTTAATCGTGT. The first number of each strain indicates the greenhouse (no. 1 to 9) where they are from. Download FIG S1, PDF file, 0.4 MB. Copyright © 2021 Zhou et al. 2021 Zhou et al. https://creativecommons.org/licenses/by/4.0/ This is an open-access article distributed under the terms of the Creative Commons Attribution 4.0 International license.

### Genotyping of A. fumigatus isolates and population genetic analyses.

**FIG 1:**
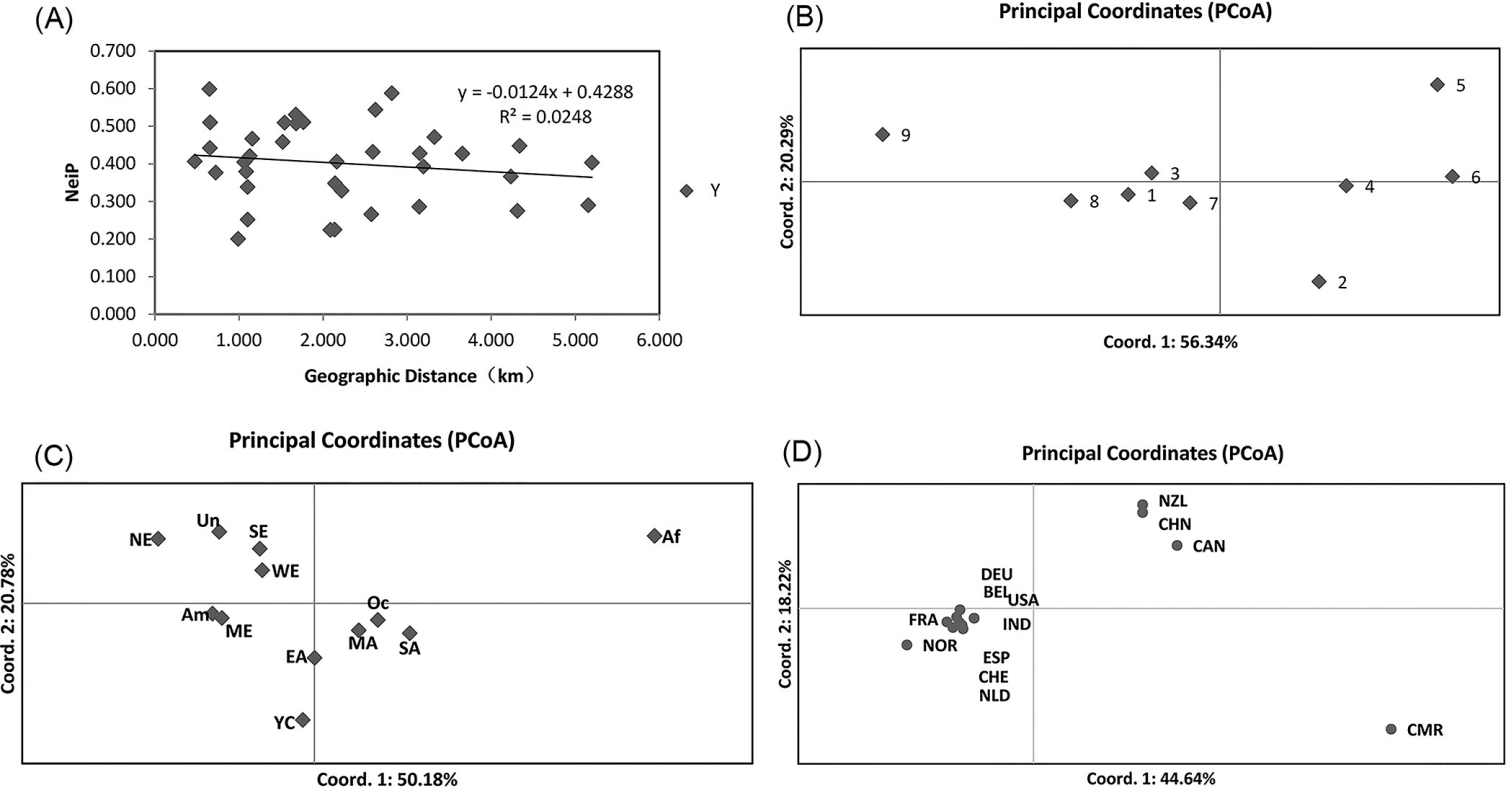
Results of Mantel test and principal-coordinate analysis. (A) Mantel test of the relationship between Nei’s genetic distance for microsatellite markers (NeiP) and geographical distance (GGD). (B) Principal-coordinate analysis of the mean population haploid genetic distance between nine geographical populations (greenhouses). (C) Principal-coordinate analysis of the mean population haploid genetic distance between 12 geographical populations. AM, America; SA, South Asia; EA, East Asia; MA, middle Asia; Af, Africa; SE, south Europe; ME, middle Europe; NE, north Europe; WE, west Europe; Oc, Oceanica; UN, unclear regions; YC, Yunnan, China. (D) Principal-coordinate analysis between 13 geographical populations in the world. NZL, New Zealand; BEL, Belgium; CAN, Canada; CMR, Cameroon; FRA, France; DEU, Germany; IND, India; NLD, Netherlands; NOR, Norway; ESP, Spain; CHE, Switzerland; USA, United States; CHN, China (Yunnan).

Of all the 233 A. fumigatus isolates from 9 different greenhouses, a total of 208 alleles and 199 multilocus genotypes were found across the nine STR loci. The number of alleles ranged from 13 to 44 per locus among the nine STR loci, with an average of 23. Among the total 208 alleles, 168 were shared between at least two of the nine greenhouses. The remain 40 alleles were found only in one greenhouse each (Table 4). The nine greenhouses also differed in their total numbers of alleles, with the largest number found in pop. 4 (115 alleles) and the smallest in pop. 3 (71 alleles). Of the total 199 multilocus genotypes, only 6 were shared by two or more greenhouses, and the other 193 were only found in one greenhouse each. Analysis of molecular variance (AMOVA) results based on clone-corrected data showed that 98% of the total genetic variation was found within individual greenhouse populations (*P* = 0.001), with a low (2%) but statistically significant genetic differentiation among the nine greenhouse populations (PhiPT = 0.019, *P* = 0.001) (see Table S2). We further investigated the extent of genetic differentiation between pairs of greenhouse populations. Among the 36 possible greenhouse population pairs, 15 pairs showed statistically significant differentiation (*P* < 0.05). The biggest differentiation was found between pop. 5 and pop. 9 (PhiPT = 0.059, *P* = 0.001), followed by that between pop. 6 and pop. 9 (PhiPT = 0.058, *P* = 0.001) (see Table S3). However, despite the observed genetic differentiation, a Mantel test showed no significant correlation between geographical distances and population genetic distances, indicating that there was gene flow between greenhouse populations of A. fumigatus but that the degree of gene flow was not correlated with their physical distances with each other (Fig. 1A) (correlation coefficient = 0.0248, *P* = 0.18).

**TABLE 4:** STR allele distributions within and among nine greenhouse populations of A. fumigatus for each of the nine STR loci

Locus	No. of alleles in the total sample	No. of alleles (no. of private alleles) in:								
		Pop. 1	Pop. 2	Pop. 3	Pop. 4	Pop. 5	Pop. 6	Pop. 7	Pop. 8	Pop. 9
STRAF2A	18	12	9	8 (1)	11 (2)	10	10	11	9	10
STRAF2B	20	11	10	8	12	8	10 (1)	12 (2)	12 (3)	8 (1)
STRAF2C	23	16	11 (2)	9	12	12 (1)	13 (1)	12 (1)	11	9
STRAF3A	44	21	16 (2)	11	21 (1)	18 (1)	21 (1)	15 (2)	13	15
STRAF3B	26	12 (1)	11 (1)	6	13	11 (2)	17 (3)	10	9	7
STRAF3C	32	15	14 (2)	11 (1)	18	13	16	14 (1)	10 (1)	10
STRAF4A	18	10	7	9	13	8	10	9	9	10 (2)
STRAF4B	14	8 (2)	7	5	9 (1)	7 (1)	5	6 (1)	5	4
STRAF4C	13	8 (1)	5	4 (1)	6 (1)	6	5	7 (2)	6	6
Total	208	113 (4)	90 (7)	71 (3)	115 (5)	93 (5)	107 (6)	96 (9)	84 (4)	79 (3)

10.1128/mSphere.00066-21.5 TABLE S2 Summary results of AMOVA within and among populations of A. fumigatus from different greenhouses and different geographic populations around the world. Download Table S2, DOC file, 0.03 MB. Copyright © 2021 Zhou et al. 2021 Zhou et al. https://creativecommons.org/licenses/by/4.0/ This is an open-access article distributed under the terms of the Creative Commons Attribution 4.0 International license.

10.1128/mSphere.00066-21.6 TABLE S3 Pairwise differentiations among nine greenhouse populations of A. fumigatus (after clone correction). Download Table S3, DOC file, 0.04 MB. Copyright © 2021 Zhou et al. 2021 Zhou et al. https://creativecommons.org/licenses/by/4.0/ This is an open-access article distributed under the terms of the Creative Commons Attribution 4.0 International license.

**FIG 2:**
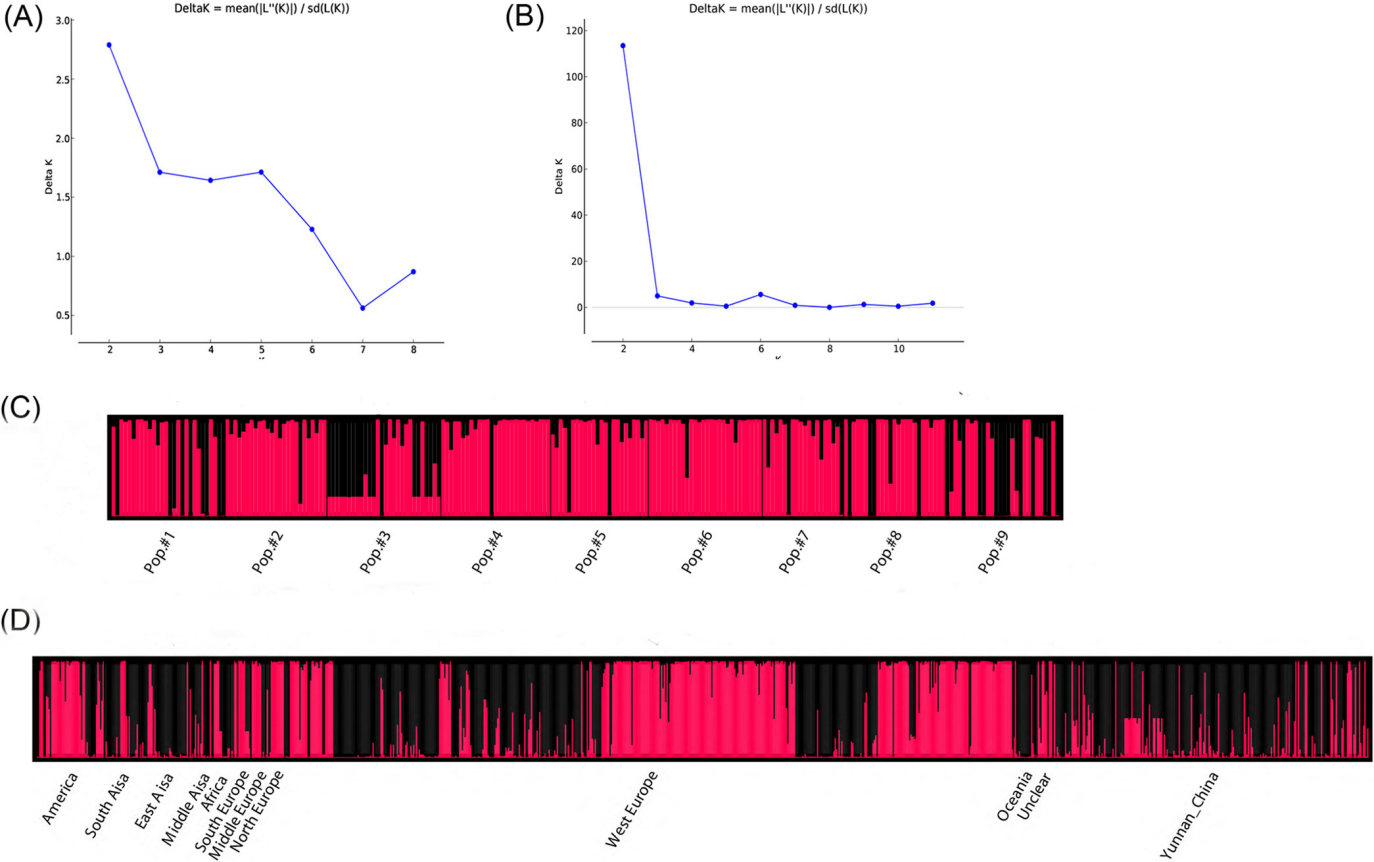
Genetic structuring results obtained from the STRUCTURE analysis. Plot of K against delta K (A) and analyses (C) for 9 greenhouse populations. Plot of K against delta K (B) and analysis (D) using 12 geographical populations from around the world, K = 2.

Results from Multilocus analyses showed limited but unambiguous evidence for recombination among the nine STR loci in the total sample, including all 233 A. fumigatus isolates (PrC = 0, *P* = 1; rBarD = 0.097441, *P* < 0.001). To identify potentially distinct genetic populations within our A. fumigatus sample, we investigated the likely number of genetic clusters using STRUCTURE software. The number of clusters (K = 2) was inferred, because the standard deviation of posterior probability was the lowest for that K (Fig. 2A and C). The conclusion was supported by clustering based on Nei’s genetic distance, with one cluster containing 13 isolates mainly from pop. 8 and the other containing 220 isolates (see Fig. S2). Principal-coordinate analysis (PCoA) using the mean pop. haploid genetic distance showed that the first principal coordinate (PC1) accounted for 56.34% of the total variance, while the second coordinate (PC2) accounted for 20.29%; these first two coordinates combined to explain 76.63% of the total variation (Fig. 1B). As can be seen from Fig. 1B, greenhouse populations 2, 5, and 9 were distinctly different from other greenhouse populations.

10.1128/mSphere.00066-21.2 FIG S2 Genetic clustering of 233 A. fumigatus isolates from greenhouses based on nine STR loci by Nei’s genetic distance for microsatellite markers. Scale bar indicates percentage identity. Download FIG S2, PDF file, 0.01 MB. Copyright © 2021 Zhou et al. 2021 Zhou et al. https://creativecommons.org/licenses/by/4.0/ This is an open-access article distributed under the terms of the Creative Commons Attribution 4.0 International license.

We further assessed the relationships between our A. fumigatus samples and those from other countries (regions). Specifically, we compared our A. fumigatus with those deposited in AfumID (https://afumid.shinyapps.io/afumID/). After clone correction, there were 876 isolates total included in our analysis. Based on their geographical origins, these isolates were divided into 12 populations (see Table S4). At the nine STR loci, there were 317 total alleles in the total sample. Of the 317 alleles, 197 alleles were shared between our greenhouse samples from Yunnan and other geographic populations. Eleven alleles were unique to our greenhouse sample (Table 5). However, there was no shared multilocus genotype between Yunnan and other geographic regions. AMOVA results of the global sample showed that 5% of total genetic variation was distributed among populations, and 95% of total genetic variation was found within populations (*P* = 0.001) (Table S2). As shown in Table S5, the pairwise comparisons showed that our greenhouse population around Kunming was significantly different from all other geographic populations, including those from East Asia (*P* = 0.001). The biggest difference between our samples and those from other areas was with those in Africa (PhiPT = 0.150, *P* = 0.001). Not surprisingly, the sample most similar to ours was from East Asia (PhiPT = 0.036, *P* = 0.001), geographically closest to our sampling sites. However, the biggest difference between any pair of geographic populations was between Africa and Northern Europe (PhiPT = 0.222, *P* = 0.001). Of the total 66 pairwise geographic population comparisons, 53 showed statistically highly significant differentiation.

**TABLE 5:** Comparison of STR alleles from Yunnan and those from 11 geographic populations of A. fumigatus from other parts of the world

Locus	No. of alleles in all 12 populations	No. of alleles in Yunnan	Specific private alleles in Yunnan	Frequency of private alleles in Yunnan
STRAF2A	20	18	8	0.0086
STRAF2B	24	20	7	0.0043
STRAF2C	28	23	7 and 36	0.0901 and 0.0043
STRAF3A	83	44	19	0.0304
STRAF3B	35	26	36 and 37	0.0086 and 0.0043
STRAF3C	48	32	38	0.0086
STRAF4A	25	18	None	None
STRAF4B	24	14	18	0.0045
STRAF4C	30	13	4 and 25	0.4206 and 0.0043
Total	317	208	11	

10.1128/mSphere.00066-21.7 TABLE S4 Geographic information of strains of A. fumigatus from other regions in comparisons with the greenhouse populations from Yunnan, China. Download Table S4, DOC file, 0.04 MB. Copyright © 2021 Zhou et al. 2021 Zhou et al. https://creativecommons.org/licenses/by/4.0/ This is an open-access article distributed under the terms of the Creative Commons Attribution 4.0 International license.

10.1128/mSphere.00066-21.8 TABLE S5 Pairwise differentiations between regional populations of A. fumigatus from different parts of the world. PhiPT values are shown below diagonal. Probability, P (rand ≥ data) based on 999 permutations is shown above diagonal. Download Table S5, DOC file, 0.04 MB. Copyright © 2021 Zhou et al. 2021 Zhou et al. https://creativecommons.org/licenses/by/4.0/ This is an open-access article distributed under the terms of the Creative Commons Attribution 4.0 International license.

STRUCTURE analyses of the global sample showed that the optimal number of genetic clusters for 876 global A. fumigatus strains was also 2, with the log likelihood of data (delta K) breakpoint appearing at a K of 2 (delta K = 143.4766) (Fig. 2B). As can be seen from Fig. 2D, there is evidence of allele sharing between these two genetic clusters (I and II). Genetic clustering based on Nei’s genetic distance also identified two broadly divergent clades, and most of our samples clustered together and formed subbranches within the two large clades (see Fig. S3). Taken together, both structure and genetic distance clustering analyses indicate that there are two distinct genetic clusters in the global sample of A. fumigatus and that both genetic clusters are broadly distributed, with different geographic populations containing different frequencies of strains in the two genetic clusters.

10.1128/mSphere.00066-21.3 FIG S3 Genetic clustering of 983 A. fumigatus isolates from 12 geographical populations based on nine STR loci by *Nei*’s genetic distance for microsatellite markers. Download FIG S3, PDF file, 2.5 MB. Copyright © 2021 Zhou et al. 2021 Zhou et al. https://creativecommons.org/licenses/by/4.0/ This is an open-access article distributed under the terms of the Creative Commons Attribution 4.0 International license.

**FIG 3:**
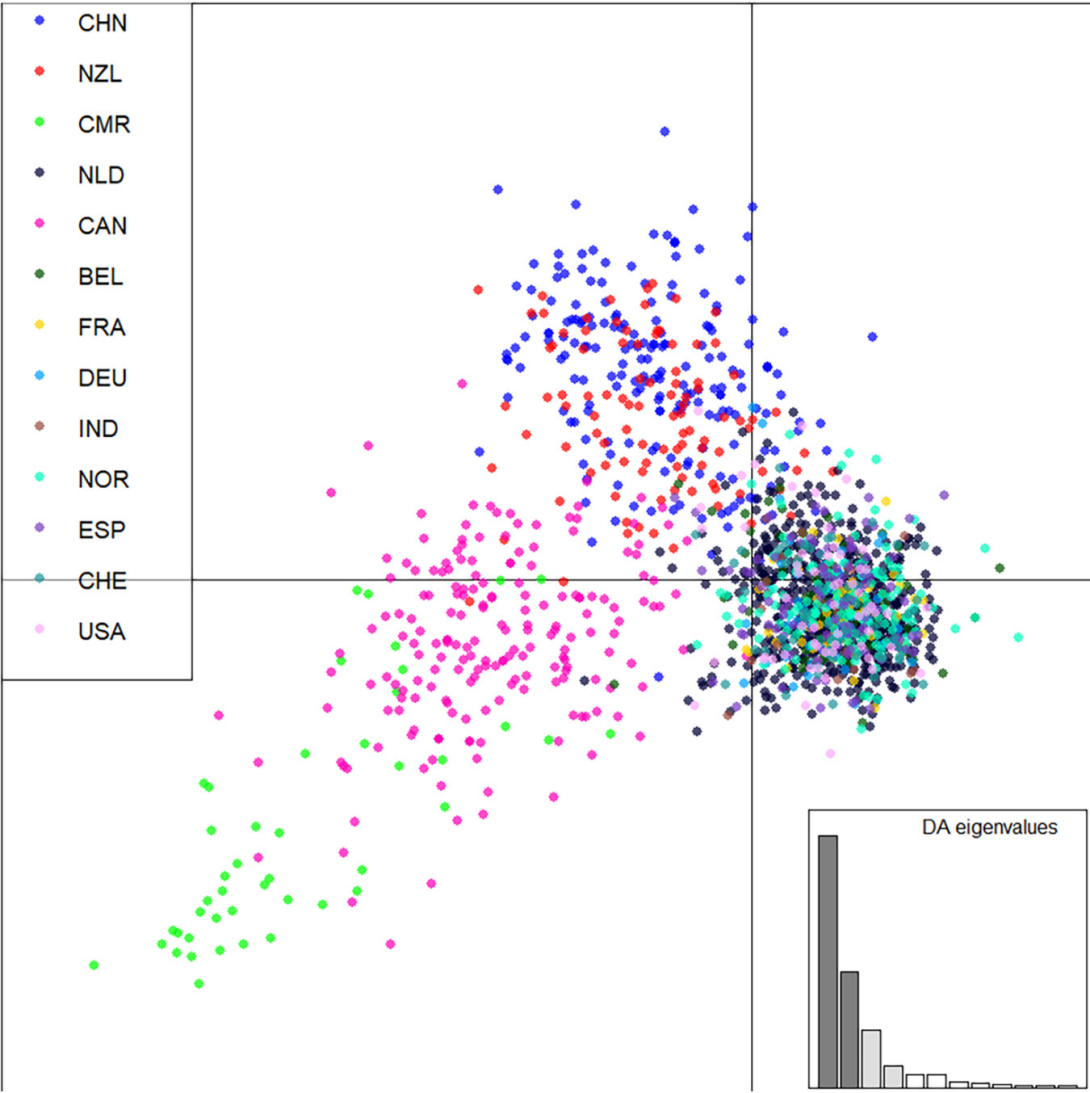
DAPC analysis between 13 geographical populations, including New Zealand, Belgium, Canada, Cameroon, France, Germany, India, Netherlands, Norway, Spain, Switzerland, United States, and China (Yunnan).

PCoA results also supported that our Yunnan population (YC) was different from most other geographic populations (Fig. 1C). However, when populations from Canada, Cameroon, and New Zealand were included in the analysis, our samples showed a greater similarity with them than with the others (Fig. 1D). Furthermore, discriminate analysis of principal components (DAPC) with the complete data set of the nine locus STR genotypes of our isolates in this study and the global data from previous studies (1, 47, 56) identified clear delineations among several regions, similar to the PCoA results (Fig. 3). Together, these results suggest both shared and unique genetic elements in the greenhouse population of A. fumigatus from Yunnan.

**FIG 4:**
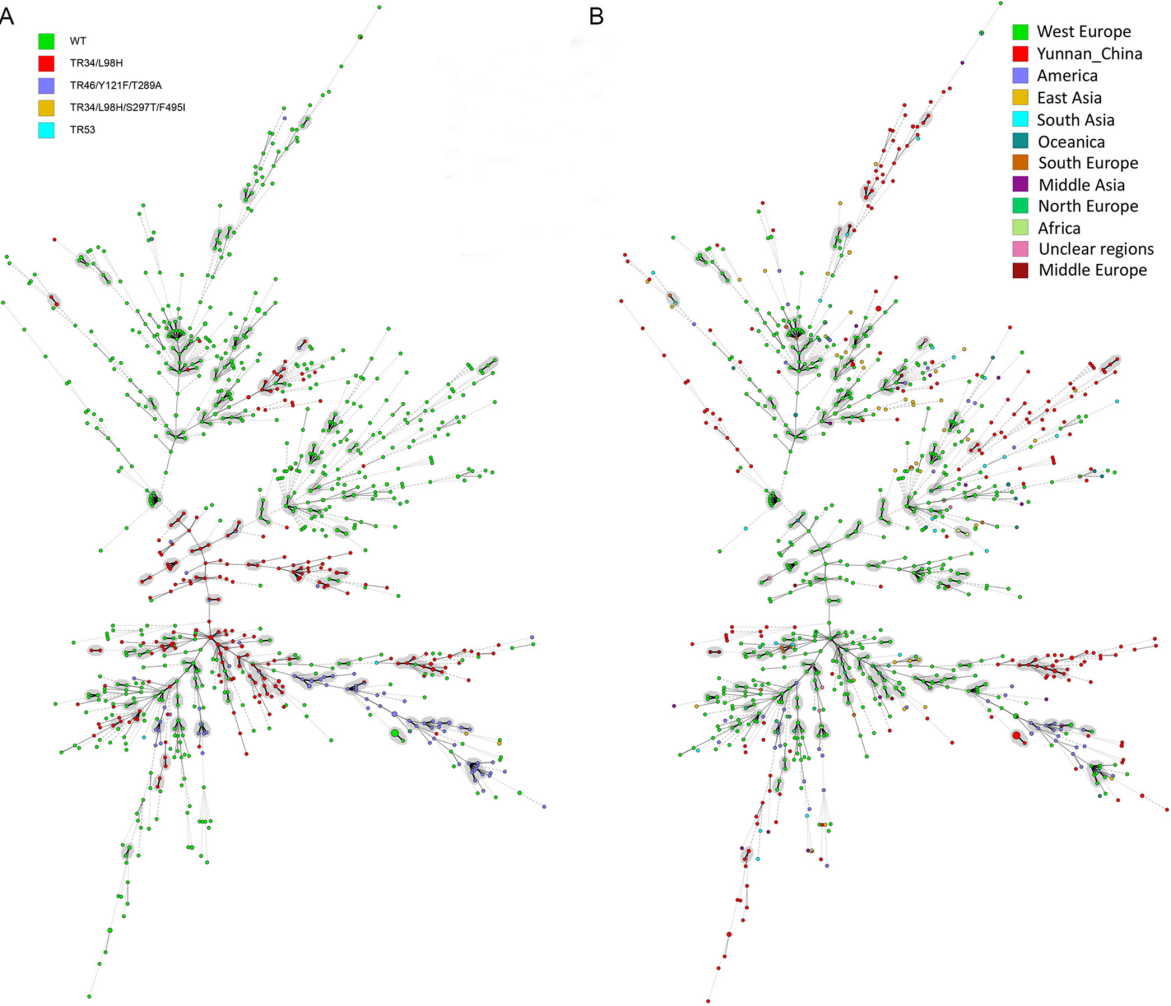
Minimum spanning tree (MST) showing the genotypic relationship between the azole-resistant isolates of various *cyp51A* genotypes and azole-susceptible A. fumigatus isolates (A) and among different geographical populations (B). Each circle corresponds to a unique genotype, and the size of the circle proportionally represents the number of isolates with that genotype. Connecting lines correspond to the number of differences between the genotypes. Short bold line, 1 difference; black line, 2 differences; long gray line, 3 differences; dotted line, 4 or more differences.

To better visualize the relationships among geographic samples and the distributions of azole resistance-associated mutations in the *cyp51A* gene, we first constructed the genotype relationships among a global collection of 983 A. fumigatus strains based on their STR genotypes, including the greenhouse strains from Yunnan (Fig. 4). Superimposed on the STR genotype relationships are azole resistance-related mutations at *cyp51A* (Fig. 4A) and the geographic locations of these strains (Fig. 4B). Our analyses show that strains with the of azole-resistant mutation TR_34_/L98H are widespread across many STR genotype groups that are separated by wild-type *cyp51A* sequences. The result is consistent with the hypothesis of multiple origins of this mutation (Fig. 4A). A similar pattern was observed for the mutant allele TR_46_/Y121F/T289A (Fig. 4A). Figure 4B highlights the geographic locations of these genotypes. The results show that the Yunnan greenhouse strains are distributed in many STR genotype groups, consistent with its diverse origins. However, several subgroups consist of strains almost entirely of those from Yunnan. Together, our results indicate both shared and independent evolution of the Yunnan greenhouse A. fumigatus populations.

### Triazole pesticide residues in soil samples and relations to azole susceptibilities.

Except for the absence of TRI fungicide in pop. 2, where resistant strains to VOR have not been found, all three triazole fungicides (TRI, TEB, and DIF) commonly used in agriculture were detected in the soil of all nine surveyed greenhouses. The concentrations of the three triazole pesticides in soil varied greatly among the nine greenhouses, ranging from 0.00088 to 0.19043 mg/kg, 0.00251 to 0.34515 mg/kg, and 0.00328 to 0.14293 mg/kg, respectively (Table 2). Statistical analysis showed that the concentrations of TRI, TEB, and DIF residues in the soil samples from each greenhouse populations were not correlated with (i) the frequencies of azole-resistant strains in each greenhouse population, (ii) the frequencies of *cyp51A* mutations in each greenhouse population, (iii) the genetic differentiations between pairs of greenhouse populations based on the nine STR loci, or (iv) the genetic differentiations between pairs of greenhouse populations based on sequences at the *cyp51A* gene (Table 6). Similarly, the genetic differentiations between pairs of greenhouse populations based on the nine STR loci and that of sequences at the *cyp51A* gene also have no correlation. However, we found a strong positive correlation between the frequency of mutations of the promoters of the *cyp51A* gene and that of VOR resistance among the nine greenhouses (Table 6), and strains with mutations TR_34_/L98H or TR_46_/Y121F/T289A had a higher voriconazole resistance level than those without the mutations in our study.

**TABLE 6:** Summary of correlations between the concentrations of fungicide residues in soil and the frequencies of medical triazole resistance, the frequency of mutations at the *cyp51A* gene, and the degrees of genetic differentiation based on STR and *cyp51A* genotypes among the nine greenhouse populations of A. fumigatus

Analyzed pairs of quantitative traits	Mantel results		Pearson correlation results by SPSS	
	Correlation coefficient	*P* value	Pearson correlation coefficient	*P* value
Concn of TRI and frequency of ITR resistance	0.412	0.13	−0.508	0.163
Concn of TRI and frequency of VOR resistance	−0.147	0.38	−0.299	0.435
Concn of TRI and frequency of cross-resistance	−0.151	0.39	−0.291	0.447
Concn of TRI and frequency of *cyp51A* mutation	−0.186	0.19	−0.322	0.398
Concn of TRI and genetic differentiation of STR	−0.127	0.54	NA^*a*^	NA
Concn of TRI and genetic differentiation of *cyp51A*	−0.011	0.67	NA	NA
Concn of TEB and frequency of ITR resistance	−0.135	0.34	0.339	0.372
Concn of TEB and frequency of VOR resistance	−0.184	0.3	−0.038	0.923
Concn of TEB and frequency of cross-resistance	−0.171	0.3	−0.032	0.936
Concn of TEB and frequency of *cyp51A* mutation	−0.175	0.18	−0.244	0.527
Concn of TEB and genetic differentiation of STR	0.102	0.17	NA	NA
Concn of TEB and genetic differentiation of *cyp51A*	−0.017	0.81	NA	NA
Concn of DIF and frequency of ITR resistance	−0.155	0.23	0.287	0.454
Concn of DIF and frequency of VOR resistance	0.000	0.63	0.201	0.604
Concn of DIF and frequency of cross-resistance	0.006	0.34	0.199	0.607
Concn of DIF and frequency of *cyp51A* mutation	−0.07	0.51	0.1	0.798
Concn of DIF and genetic differentiation of STR	−0.12	0.74	NA	NA
Concn of DIF and genetic differentiation of *cyp51A*	−0.115	0.3	NA	NA
Genetic differentiation of STR and that of *cyp51A*	0.222	0.18	NA	NA
Frequency of VOR resistance and that of *cyp51A* mutation	0.955	0.01	0.960	0.000
Frequency of cross-resistance and that of *cyp51A* mutation	0.911	0.01	0.960	0.000

a NA, not available.

## DISCUSSION

### High genetic diversity and gene flow.

In this study, high-level genetic diversity was found among the 233 A. fumigatus strains from nine greenhouses within Jinning county, metropolitan Kunming, China. The high-level genetic diversity included a number of novel alleles and many novel genotypes not reported previously in early investigations. Our results showed that the overall genetic difference among the greenhouse populations around Kunming were very limited (PhiPT = 0.019, *P* = 0.001), similar to results reported recently from six local populations from Auckland, New Zealand (47). The limited differentiation is consistent with gene flow between local greenhouse populations, potentially mediated by air current, anthropogenic activities, and/or shared population histories. Indeed, previous studies have shown that certain multilocus genotypes were shared by isolates from different ecological types (air, water, and oil) or even countries and continents separated by long distances (1, 5). However, different from the Auckland, New Zealand, population where no genetic differentiation was found, several greenhouse populations here showed significantly different allele frequencies from each other. The results suggest that the greenhouse structure could act as gene flow barriers among greenhouses, causing the frequencies of certain alleles and allelic combinations to differ from each other among the greenhouses.

While a few multilocus genotypes were shared among isolates from the nine greenhouses, no genotype sharing was found between our isolates and those from AfumID representing broad geographic regions in the world. DAPC and PCoA results showed that our isolates were clustered differently from most other geographic populations. Many factors could have contributed to the observed geographic differentiation, including geographic barriers, nonrandom mating, mutation, genetic drift, and selection (1, 5, 46, 47). Furthermore, sample sizes could also affect the observed differentiation between geographical populations (56). Yunnan is characterized by high mountains and deep rivers, and exchanges of goods and travel between Yunnan and other regions were relatively limited until recently. These factors could have contributed to the observed differentiation between our greenhouse samples of A. fumigatus and those from other geographic regions.

Among the alleles in our A. fumigatus isolates, 98 of 233 isolates had allele 4 at locus 4C, representing a very high frequency (0.42) in our sample. Interestingly, this allele was also found in high frequency (0.431) in Cameroon, Western Africa, but is absent in strains from nine other geographic populations worldwide (Belgium, France, Germany, India, Netherlands, Norway, Spain, Switzerland, and the United States) (56). At present, the reason(s) for the shared high frequency of this allele between the two distant geographic populations in southwestern China and Cameroon is unknown.

STRUCTURE analyses of both our greenhouse strains and a global collection of A. fumigatus strains identified two broadly divergent clades (5, 25, 31, 39–42). This result contrasts with what was described previously based on a global sample of 2,026 A. fumigatus isolates from 14 countries in five continents, where nine genetic clusters were identified (1, 47, 56). We believe that the reduction in the number of genetic clusters was most likely due to the increased sample sizes representing more geographic regions and with more genotypes. These new genotypes linked a previously reported large number of small clusters into a small number of large clusters (i.e., natural reproductively distinct groups). However, the current number of two distinct clusters in this species is likely robust, as it has received support not only from the nine STR loci but also from whole-genome sequence analyses (57), potentially representing two different genome species (58).

### Azole resistance and correlation with soil fungicide residues and *cyp51A* mutations.

In our study, we found a large number of medical triazole-resistant strains from the nine greenhouses. Specifically, almost 80% of all strains were resistant to one of the two commonly used medical triazole drugs (ITR and VOR) for treating invasive aspergillosis, with 33% showing cross-resistance to both ITR and VOR. The frequency of triazole resistance in our sample represents the highest so far reported in the literature across the globe. For example, Chen et al. (43) reported that the prevalence of azole resistance from hospital gardens, city parks, and farmlands in China was 1.4% (2/144). The prevalence of azole resistance from greenhouses in Zhejiang Province in China was 4.11% (3/73) (27), that from strawberry fields was 10.20% (21/206) (43). Outside China, the prevalence of azole resistance was approximately 19% in the United States (17), approximately 30% in the Netherlands (22), and approximately 19% in Italy (24). Within Italy, the highest rate of azole resistance was found in apple orchards (50% [3/6]) and olive groves (41% [7/17]).

Greenhouse environments insulate crops from the influence of natural growth conditions and seasonal fluctuations of weather conditions. To maintain high productivity year-round in such an environment, frequent applications of fungicides are a common practice in China. For example, it has been estimated that the total amount of azole fungicides used in agriculture in China was more than 27 million kg/year during 2013 to 2016, and the usage of TEB and prochloraz almost doubled between 2012 and 2016 (43). The detection of the three common azole fungicides in the soil of almost all nine greenhouses is consistent with their frequent usage in these greenhouses. Specifically, the azole residue concentrations of DIF and TEB in our greenhouse soils were much higher than those in agricultural soil from other parts of China (Harbin, Beijing, Weifang, Nanjing, Wuhan, Hangzhou, Yichun, and Loudi), with concentrations of DIF and TEB that range from 0.0104 to 0.0385 mg/kg and 0.015 to 0.0805 mg/kg, respectively (43). Frequent applications of azole fungicides would select for fungal strains that are resistant to these drugs. Due to the high structural and functional similarities between agricultural and medical triazoles, resistance to agricultural triazole often leads to resistance to medical triazoles (59). An additional factor is the high-temperature environment in greenhouses and tolerance of A. fumigatus to high temperature. Kunming’s monthly average temperature ranges from 12 to 22°C; however, greenhouses can reach very high temperatures, including in winter during midday. At a high temperature such as 45°C, many fungi in the greenhouse environment would have reduced survival and reproduction. However, A. fumigatus can grow well at temperatures above 50°C, thus potentially contributing to its high prevalence in the greenhouse environment. Its high population size in greenhouses increases the likelihood that mutations to azole resistance could develop and be selected due to the presence of azole fungicides in the soil.

Thirty-three of the 233 A. fumigatus isolates had insertional mutations in the promoter region of the *cyp*5*1A* gene. Three types of insertional mutations were detected: TR_34_ (*n* = 28), TR_46_ (*n* = 3), and TR_53_ (*n* = 2). All three types of mutations have been reported previously in other geographic areas such as Europe, India, East Asia, the Middle East, and the Americas (19, 42, 60–63). However, most of these insertional mutations were also associated with amino acid substitution mutations, similar to what we found here. For example, the most frequent mutation found in our study, TR_34_/L98H (*n* = 25; 76%), was also the most frequently found in India and the Netherlands (47, 60). A mutation found in our sample, TR_46_/Y121F/T289A (*n* = 3; 9%), was the most frequently found in flower fields in Colombia (*n* = 17; 80.9%), where the proportion of TR_34_/L98H accounted for only 4.8% (1/21) of the azole-resistant isolates (64). Interestingly, experimental exposure of A. fumigatus to the agricultural fungicide TRI led to reduced susceptibility to three clinical triazole drugs (ITR, VOR, and POS) and with the most common mutation being TR_46_/Y121F/T289A (*n* = 6), but no TR_34_/L98H mutation was found (27). Compared to the TR_34_ and TR_46_ insertional mutations, the TR_53_ insertional mutation was rarely reported (only twice) in the past few years. The first report of the TR_53_ mutation was in 2009, and the mutated sample was isolated from a patient with chronic granulomatous disease in the Netherlands (23). The second report was in 2016, and two strains with the mutations were isolated from flower fields in Colombia (64). To our knowledge, our study is the first report of the TR_53_ mutation from Asia. In addition, our analyses of STR genotypic relationships among global strains indicated that different mutations of the *cyp51A* gene could lead to triazole resistance and that the same type of mutation could arise independently among strains of different genetic backgrounds in different geographic regions. As shown previously, some of the triazole-resistant mutant genotypes are capable of dispersing long distances (59).

Triazole susceptibility tests showed that except for one TR_34_/L98H isolate that was sensitive to VOR, all other 32 A. fumigatus isolates with the TR_34_ insertional mutation were resistant to both ITR and VOR (MIC ≥ 4 μg/ml) and had high tolerance to TRI (MIC ≥ 32 μg/ml) and TEB (MIC ≥ 16 μg/ml). These results indicated that cross-resistance was frequent in our strains and attention should be paid to designing alternative treatment strategies in the clinic when patients are infected with multidrug-resistant fungal pathogens. Interestingly, the 33 A. fumigatus isolates with insertional mutations were not evenly distributed among the nine greenhouses, with frequencies ranging from 0% (pop. 2) to ∼40% (pop. 1 and pop. 9). As shown in pop. 2, while the presence of azole fungicides is likely a significant selection force for azole-resistant strains in the environment (43, 59, 65, 66), we failed to identify a statistically significant positive correlation between the detected azole residue concentration in the greenhouse soil and the prevalence of azole-resistant strains and mutations among the nine greenhouses. A similar result was obtained in a previous study between azole resistance of A. fumigatus and azole usage in Italy (67). At present, the reason for such a lack of correlation is not known. In our study, only one-time testing of the soil samples for both the fungus and the azole residues was performed for each greenhouse. Multiple testing over a prolonged period of time of both the fungus and the soil samples is needed in order to critically evaluate their relationships (68).

Our statistical analysis showed a strong positive correlation between the frequency of insertional mutations in the promoter of the *cyp51A* gene and that of VOR resistance among the 9 greenhouses (*R* = 0.960, *P* < 0.001). However, the overall frequency of insertional mutations and associated SNPs (0.1416 [33/233]) was significantly lower than those of ITR resistance (0.7854 [183/233]) and VOR resistance as determined based on microbroth susceptibility testing (0.3391 [79/233]). These results confirmed that, though very important, mutations at the *cyp51A* gene are not the only mechanisms responsible for azole resistance in these greenhouses. Mutations in other parts of the *cyp51A* gene independent of the insertional mutations and in other genes such as those encoding ABC transporters, MFS transporters, and 3-hydroxy-3-methylglutaryl-coenzyme A (HMG-CoA) and those involved in stress response and biofilm formation could be involved as well. For example, we found that there was a high level of polymorphism in the coding DNA sequence (CDS) of the *cyp51A* gene, with 34.76% (81/233) of our A. fumigatus isolates containing SNPs different from the reference strain, including 4 synonymous mutations and 11 nonsynonymous mutations. Natural selection drives adaptive evolution by selecting for and increasing the occurrence of beneficial traits in a population that enhance the individual’s ability to survive in the environment (69). In our study, there was evidence that mutations in the *cyp51A* gene was positively selected, consistent with azoles playing a role in the origin and maintenance of these mutations (66).

Our phylogenetic analysis of the *cyp51A* gene sequences suggested that the azole resistance-associated nonsynonymous mutations on *cyp51A* gene did not share ancestry but arose multiple times independently in the natural populations. Interestingly, we found that among the 4 synonymous mutation sites, the mutation of 267A→G, 1074G→A, and 1362C→T occurred together at a frequency of 0.0601 (14/233), but evidence for recombination/convergent mutation was found between two pairs of sites (267A→G and 540G→A; 540G→A and 1074G→A). Our study is the first report of evidence for potential recombination within the *cyp51A* gene in A. fumigatus. Previous studies have shown that recombination could accelerate adaptation to novel environmental conditions in a related fungus Aspergillus nidulans (25, 70). Recombination-mediated adaptive evolution could similarly occur in A. fumigatus. In contrast, the coexistence of amino acid substitutions at several sites across the population at high frequencies suggested that such combinations likely have functional significance.

In summary, our study identified novel allelic and genotypic diversities in greenhouse populations of A. fumigatus in a small region from southwestern China. Significantly, we found a high frequency (∼80%) of triazole-resistant strains, the highest reported so far in the literature. Many of those strains have high tolerance to multiple azole drugs, including both agricultural fungicides and medical triazoles used to treat patients with invasive *Aspergillus* infections. Our study calls for systematic evaluation of the effects of azole fungicide usage in greenhouses on human health. More broadly, with increasing prevalence of infections by A. fumigatus and other fungal pathogens, systematic studies linking agricultural and environmental fungicide usages and drug-resistant fungal infections are urgently needed.

## MATERIALS AND METHODS

### Soil samples.

In December 2019, 900 soil samples from nine different greenhouses were collected at a depth of 0 to 5 cm. These greenhouses were located in Jinning county, part of metropolitan Kunming, the capital city of Yunnan Province in southwest China. These greenhouses were used for the productions of coriander, cucurbita pepo, pea, lettuce, and fennel. Within each greenhouse, 100 soil samples of approximately 10 g each were collected in sterile zip-lock plastic bags. Individual soil samples were approximately 2 m apart from each other. The nine greenhouses were approximately 0.5 to 5.0 km away from each other. Detailed information of the soil samples is shown in Table 2.

### Strain isolation, DNA extraction, molecular identification, and STR genotyping.

Isolation of A. fumigatus complex strains was according to previously described methods (47). To minimize isolating strains of the same genotype and phenotype, we obtained and analyzed a maximum of one isolate from each soil sample. We used the tip of a very thin needle to transfer spores and hyphae from the edge of a colony into a tube containing sterile distilled water, vortexed the spore suspension, and streaked the suspension onto a fresh medium plate. A single newly formed colony distant from other colonies on the plate was then transferred to another new plate from which strain phenotype and genotype were subsequently determined for each strain. Genomic DNA was extracted from the mycelia collected from single-spore cultures growing on a cellophane membrane on peptone-dextrose agar (PDA) medium according to the modified cetyltrimethylammonium bromide (CTAB) method (71).

ITS sequences of 60 randomly selected strains representing all populations were obtained according to the method described by Zhang et al. (72) to further confirm the morphological identification of these A. fumigatus isolates. Genotyping of A. fumigatus isolates was performed with a panel of nine STR loci (namely, STR*Af* 2A, 2B, 2C, 3A, 3B, 3C, 4A, 4B, and 4C), as previously described (38). We determined the number of microsatellite repeats at each locus for each strain. Alleles at the nine STR loci were combined to generate the multilocus STR genotype for each strain.

### Population genotype data analyses.

Aside from our own data obtained for the nine greenhouses in Jining, Kunming, the STR genotype data of A. fumigatus isolates from other countries, which were reported in previous work and deposited in the STR*Af* database (http://afumid.shinyapps.io/afumID) (1, 5, 47), were used for comparative analyses. The STR genotype data of our samples was imported into GenAlEx 6.1 (73) to calculate the pairwise PhiPT values between pairs of populations from each greenhouse and determine the potential correlation between genetic and geographical distances (Mantel test) among populations from the greenhouses. The analysis of molecular variance (AMOVA) was performed to estimate the relative contributions of geographic separation to the overall genetic variation. To investigate the pattern of genetic diversity in our data, a multivariate analysis was first conducted via discriminate analysis of principal components (DAPC), which was implemented by adegenet package in R version 3.0 (Vienna, Austria) to detect the relationship between our A. fumigatus isolates and those from other countries (74), including those of our own data from Canada, Cameroon, and New Zealand reported in our previous studies (1, 47, 56). Using the LOCPRIOR model with admixture and correlated allele frequencies, the program STRUCTURE version 2.3.3 (75) was then used to explore the number of genetic clusters (K) occurring in the combination of our samples and all 750 STR*Af* genotypic data downloaded from http://afumid.shinyapps.io/afumID. A total of 10 replicates were performed of each simulation for the number of genetic clusters K of 1 to 12, with a burn-in of 10,000, and Markov chain Monte Carlo (MCMC) of 100,000 iterations for the best estimate of K. Unweighted pair group method using average linkages (UPGMA) clustering was conducted to investigate the genetic relationship between geographic populations and multiple *cyp51A* variants of our A. fumigatus isolates and those from AfumID (750 isolates). A minimum spanning tree was constructed with default settings for microsatellite markers (BIONUMERICS 8.0; Applied Maths, Belgium).

### Antifungal susceptibility of A. fumigatus isolates.

Susceptibilities of each strain to four triazoles, ITR, VOR, TRI, and TEB, were tested according to the CLSI M38-A2 method (76). Triazoles ITR and VOR are commonly used for treating patients with invasive fungal infections, including IA, while TRI and TEB are frequently used in agricultural fields, including those for growing vegetables. The MIC was defined as the lowest antifungal concentration at which visual growth of a microorganism was completely inhibited. MIC_50_ was defined the lowest concentration that inhibited 50% of visual growth. There is currently no recommended cutoff value for defining resistance for TRI and TEB fungicides. Instead, our methods were in accordance with the study by Chowdhary et al. (60), and we described the concentration range of these two drugs used in our tests and their corresponding MIC values as recommended by for CLSI M38-A2 method. The tested drug concentration ranges of ITR, VOR, TRI, and TEB were 0.0078 μg/ml to 16 μg/ml, 0.0078 μg/ml to 16 μg/ml, 0.0156 μg/ml to 32 μg/ml, and 0.0156 μg/ml to 32 μg/ml, respectively. A. fumigatus isolates were grouped as susceptible (MIC ≤ 1 μg/ml) and resistant (MIC ≥ 4 μg/ml) for ITR and VOR (76). Reference strains ATCC 22019 (Candida parapsilosis), ATCC 22019 (Candida krusei), and ATCC 10231 (Candida albicans) were used as controls. Our susceptibility tests were repeated for each strain at least three times on different days, including on all reference strains and our susceptible and resistant A. fumigatus strains.

### *cyp51A* gene sequencing and analyses.

Primers A7 (5′-TCATATGTTGCTCAGCGG-3′) and P450-A2 (5′-CTGTCTCACTTGGATGTG-3′) were used to amplify the *cyp51A* gene and its promoter region from each strain, followed by DNA sequencing, using the procedures described previously (77). Mutations of *cyp51A* gene and its promoter region were identified by comparing with the reference sequence of a wild-type azole-susceptible A. fumigatus strain under the accession number AF338659 in GenBank (77, 78). A maximum likelihood (ML) phylogeny based on synonymous sites of *cyp51A* gene was constructed using raxmlGUI1.3 with 1,000 replicates (79). The ancestral range of the genotypes of nonsynonymous sites of *cyp51A* was reconstructed using dispersal-vicariance analysis (S-DIVA) implemented in the program Reconstruct Ancestral State in Phylogenies (RASP) 4.2 (80).

To identify a potential convergent mutation(s) and recombination within the *cyp51A* gene, we also assessed the associations among single nucleotide polymorphic sites, using the program Multilocus 1.3. Specifically, two tests were performed for our data, namely, the index of association (I_A_) and phylogenetic compatibility (81). Both analyses were performed for synonymous and nonsynonymous SNPs. Since the numbers of SNPs were very different among greenhouse samples (see Results), the I_A_ values were adjusted based on the numbers of variable nucleotide sites to obtain the standardized rBarD values. Finally, to infer the potential selection on the observed sequence variation, mutational rate ratio ω (*dN/dS*) at the *cyp51A* locus was tested using MEGA version 6 (82). The ratio of nonsynonymous to synonymous mutational rates was calculated to infer the types of selection impacting specific sites, with ω between 0 and 1, equal to 1, and >1 representing negative purifying selection, neutral evolution, and positive selection, respectively (83).

### Detection of triazole fungicide residues in soil samples.

In this study, three main fungicides (TRI, TEB, and DIF) used in agriculture in China were selected as targets for triazole pesticide residue detections in the greenhouse soils. Gas chromatography-mass spectrometry (GC-MS) with a GCMS-QP2020 (Shimadzu, Japan) was employed, and known reference solution concentrations (100 μg/ml; TMRM, China) were used as controls. A total of nine soil samples were tested, one from each greenhouse. To prepare the soil sample from each greenhouse, 2 g of soil from each of the 100 soil samples from each greenhouse were combined to make one bulk soil sample of 200.0 g.

Each soil sample (200.0 g) was ultrasonically extracted with 200 ml of acetonitrile and 10.0 g NaCl for 30 min, and then the supernatant was transferred to the rotary evaporator for condensation. One hundred fifty milliliters acetonitrile was added to the soil residue again, and they were extracted twice. After evaporation and extraction, the concentrated solution was cleaned using a series of purification columns (Mega Bond Elut-NH2 and Mega BE Carbon/NH2; Agilent Technologies, USA). The liquid was collected and condensed again to approximately 0.5 ml in a water bath at a temperature of 40°C. Five milliliters *n*-hexane was added to the concentrated solution twice to carry out solvent exchange. After the final volume of the liquid reached approximately 1 ml, 40 μl of an internal standard solution was added and mixed. Soil samples from each greenhouse were tested twice.

### Correlation analysis.

Correlation analysis is used to detect the potential relationships among triazole fungicide residues, mutation frequency at the *cyp51A* gene, and the occurrence of ARAF among the greenhouses. Moreover, the relationship between genetic differentiation of nine STRs and that of *cyp51A* gene from isolates in each greenhouse population was also calculated. IBM SPSS statistical software V22.0 was used for the correlation analysis.

### Data availability.

All data necessary to support the conclusions of the study are contained in the figures, tables, and supplemental files associated with the manuscript.
