# Supplementary material for: Extensive Genetic Diversity and Widespread Azole Resistance in Greenhouse Populations of Aspergillus fumigatus in Yunnan, China
Source: mSphere. 2021 Feb 10;6(1):e00066-21. doi: 10.1128/mSphere.00066-21 (PMC8544883; doi:10.1128/mSphere.00066-21)
Supplement: TABLE S1 [file msphere.00066-21-st001.doc]

Table S1 Azole-susceptibility profiles and insertional mutation in the promoter region of the *cyp51A* gene in *A. fumigatus* isolates.

| Pop | Strain  code | MIC（µg/mL） | | | | Promoter  mutation type |
| --- | --- | --- | --- | --- | --- | --- |
|  |  | itraconazole | voriconazole | triadimefon | tebuconazole |  |
| Pop.#1 | 1-1 | >16 | >16 | >32 | >32 |  |
| Pop.#1 | 1-10 | >16 | 16 | >32 | >32 | TR 34 /L98H |
| Pop.#1 | 1-11 | >16 | >16 | >32 | >32 |  |
| Pop.#1 | 1-12 | >16 | 8 | >32 | 32 |  |
| Pop.#1 | 1-13 | 4 | 8 | >32 | >32 |  |
| Pop.#1 | 1-14 | >16 | >16 | >32 | 32 |  |
| Pop.#1 | 1-15 | >16 | >16 | >32 | >32 |  |
| Pop.#1 | 1-16 | 4 | 16 | >32 | >32 |  |
| Pop.#1 | 1-17 | >16 | 8 | >32 | >32 |  |
| Pop.#1 | 1-18 | >16 | 1 | >32 | 32 |  |
| Pop.#1 | 1-19 | >16 | 4 | >32 | 4 |  |
| Pop.#1 | 1-2 | >16 | 8 | >32 | >32 |  |
| Pop.#1 | 1-20 | >16 | 2 | >32 | 4 |  |
| Pop.#1 | 1-21 | >16 | 4 | >32 | 32 |  |
| Pop.#1 | 1-22 | >16 | 4 | >32 | >32 | TR 34 /L98H |
| Pop.#1 | 1-23 | >16 | 16 | >32 | >32 | TR 34 /L98H |
| Pop.#1 | 1-24 | >16 | 8 | >32 | >32 |  |
| Pop.#1 | 1-25 | >16 | >16 | >32 | >32 | TR 34 /L98H |
| Pop.#1 | 1-26 | >16 | 8 | >32 | >32 |  |
| Pop.#1 | 1-27 | >16 | 8 | >32 | 32 | TR 34 /L98H/S297T/F495I |
| Pop.#1 | 1-28 | 16 | 1 | >32 | >32 |  |
| Pop.#1 | 1-3 | >16 | >16 | >32 | >32 | TR 46 /Y121F/T289A |
| Pop.#1 | 1-4 | >16 | >16 | >32 | 32 | 53bp |
| Pop.#1 | 1-5 | >16 | 8 | >32 | 32 | TR 34 /L98H |
| Pop.#1 | 1-6 | >16 | 16 | >32 | >32 | 53bp |
| Pop.#1 | 1-7 | >16 | 2 | >32 | 4 |  |
| Pop.#1 | 1-8 | >16 | >16 | >32 | >32 | TR 34 /L98H |
| Pop.#1 | 1-9 | >16 | >16 | >32 | >32 | TR 34 /L98H |
| Pop.#2 | 2-1 | 4 | 1 | >32 | 2 |  |
| Pop.#2 | 2-10 | 4 | 2 | >32 | 16 |  |
| Pop.#2 | 2-11 | 4 | 1 | >32 | >32 |  |
| Pop.#2 | 2-12 | 4 | 1 | >32 | 4 |  |
| Pop.#2 | 2-13 | 8 | 0.5 | >32 | 4 |  |
| Pop.#2 | 2-14 | 2 | 0.25 | >32 | 4 |  |
| Pop.#2 | 2-15 | 2 | 0.5 | >32 | 4 |  |
| Pop.#2 | 2-16 | 2 | 1 | >32 | 4 |  |
| Pop.#2 | 2-17 | 2 | 0.5 | >32 | 4 |  |
| Pop.#2 | 2-18 | 2 | 0.5 | >32 | 4 |  |
| Pop.#2 | 2-19 | 4 | 0.5 | >32 | 4 |  |
| Pop.#2 | 2-2 | >16 | 2 | >32 | 4 |  |
| Pop.#2 | 2-20 | 4 | 0.25 | >32 | 4 |  |
| Pop.#2 | 2-21 | 2 | 0.25 | >32 | 4 |  |
| Pop.#2 | 2-22 | 4 | 1 | >32 | 2 |  |
| Pop.#2 | 2-23 | 4 | 0.5 | >32 | 8 |  |
| Pop.#2 | 2-24 | 4 | 0.25 | >32 | 4 |  |
| Pop.#2 | 2-25 | 4 | 0.5 | >32 | 4 |  |
| Pop.#2 | 2-3 | >16 | 2 | >32 | 4 |  |
| Pop.#2 | 2-4 | 4 | 1 | >32 | 4 |  |
| Pop.#2 | 2-5 | 4 | 1 | >32 | 4 |  |
| Pop.#2 | 2-6 | >16 | 0.5 | >32 | 4 |  |
| Pop.#2 | 2-7 | 8 | 0.5 | >32 | 4 |  |
| Pop.#2 | 2-8 | 8 | 0.5 | >32 | 4 |  |
| Pop.#2 | 2-9 | 8 | 0.5 | >32 | 4 |  |
| Pop.#3 | 3-1 | 2 | 0.5 | >32 | 2 |  |
| Pop.#3 | 3-10 | 4 | 0.25 | >32 | 2 |  |
| Pop.#3 | 3-11 | 16 | 0.5 | >32 | 2 |  |
| Pop.#3 | 3-12 | 2 | 0.5 | >32 | 2 |  |
| Pop.#3 | 3-13 | 2 | 1 | >32 | 4 |  |
| Pop.#3 | 3-14 | >16 | 1 | >32 | 8 |  |
| Pop.#3 | 3-15 | 4 | 0.5 | >32 | 4 |  |
| Pop.#3 | 3-16 | 2 | 0.25 | >32 | 4 |  |
| Pop.#3 | 3-17 | 2 | 0.5 | 32 | 2 |  |
| Pop.#3 | 3-18 | 2 | 0.5 | >32 | 8 |  |
| Pop.#3 | 3-19 | 2 | 0.5 | >32 | 1 |  |
| Pop.#3 | 3-2 | 2 | 1 | >32 | 2 |  |
| Pop.#3 | 3-20 | 2 | 0.5 | >32 | 2 |  |
| Pop.#3 | 3-21 | 16 | 1 | >32 | 32 | TR 34 /L98H |
| Pop.#3 | 3-22 | 2 | 0.25 | >32 | 4 |  |
| Pop.#3 | 3-23 | 2 | 0.25 | >32 | 4 |  |
| Pop.#3 | 3-24 | 2 | 1 | >32 | 8 |  |
| Pop.#3 | 3-25 | 16 | 1 | >32 | 2 |  |
| Pop.#3 | 3-26 | 2 | 1 | >32 | 4 |  |
| Pop.#3 | 3-27 | 4 | 0.5 | >32 | 4 |  |
| Pop.#3 | 3-28 | 2 | 1 | >32 | 8 |  |
| Pop.#3 | 3-3 | 1 | 0.5 | >32 | 4 |  |
| Pop.#3 | 3-4 | 1 | 0.5 | >32 | 2 |  |
| Pop.#3 | 3-5 | 1 | 0.5 | 32 | 1 |  |
| Pop.#3 | 3-6 | 1 | 0.5 | 32 | 4 |  |
| Pop.#3 | 3-7 | >16 | 0.125 | >32 | 4 |  |
| Pop.#3 | 3-8 | >16 | 8 | >32 | >32 |  |
| Pop.#3 | 3-9 | 2 | 1 | >32 | 0.125 |  |
| Pop.#4 | 4-1 | >16 | 0.5 | >32 | 1 |  |
| Pop.#4 | 4-10 | 16 | 0.5 | >32 | 1 |  |
| Pop.#4 | 4-11 | 4 | 0.5 | >32 | 2 |  |
| Pop.#4 | 4-12 | 2 | 0.5 | >32 | 4 |  |
| Pop.#4 | 4-13 | 16 | 2 | >32 | 1 |  |
| Pop.#4 | 4-14 | 2 | 1 | >32 | 1 |  |
| Pop.#4 | 4-15 | >16 | 2 | >32 | 8 |  |
| Pop.#4 | 4-16 | >16 | 2 | >32 | 4 |  |
| Pop.#4 | 4-17 | >16 | 2 | 32 | 2 |  |
| Pop.#4 | 4-18 | 4 | 1 | 32 | 2 |  |
| Pop.#4 | 4-19 | 8 | 1 | 32 | 2 |  |
| Pop.#4 | 4-2 | 2 | 0.5 | >32 | 1 |  |
| Pop.#4 | 4-20 | >16 | 4 | >32 | 8 | TR 34 /L98H/S297T/F495I |
| Pop.#4 | 4-21 | >16 | 1 | 32 | 4 |  |
| Pop.#4 | 4-22 | 2 | 1 | 32 | 2 |  |
| Pop.#4 | 4-23 | 2 | 0.5 | 32 | 1 |  |
| Pop.#4 | 4-24 | 8 | 0.5 | 32 | 2 |  |
| Pop.#4 | 4-25 | 2 | 0.25 | 32 | 1 |  |
| Pop.#4 | 4-26 | 2 | 1 | 32 | 4 |  |
| Pop.#4 | 4-27 | 1 | 1 | 32 | 4 |  |
| Pop.#4 | 4-3 | 4 | 0.5 | >32 | 1 |  |
| Pop.#4 | 4-4 | 4 | 16 | >32 | 1 |  |
| Pop.#4 | 4-5 | 1 | 0.5 | >32 | 1 |  |
| Pop.#4 | 4-6 | 1 | 0.5 | >32 | 1 |  |
| Pop.#4 | 4-7 | 8 | 2 | >32 | 2 |  |
| Pop.#4 | 4-8 | 8 | 1 | >32 | 2 |  |
| Pop.#4 | 4-9 | 16 | 0.5 | >32 | 2 |  |
| Pop.#5 | 5-1 | >16 | 1 | >32 | 8 |  |
| Pop.#5 | 5-10 | 8 | 4 | >32 | 4 |  |
| Pop.#5 | 5-11 | 1 | 0.25 | >32 | 1 |  |
| Pop.#5 | 5-12 | 16 | 4 | >32 | 16 |  |
| Pop.#5 | 5-13 | >16 | 8 | >32 | 16 | TR 34 /L98H |
| Pop.#5 | 5-14 | 8 | 1 | >32 | 4 |  |
| Pop.#5 | 5-15 | 2 | 0.5 | >32 | 0.5 |  |
| Pop.#5 | 5-16 | 4 | 2 | >32 | 2 |  |
| Pop.#5 | 5-17 | 2 | 2 | >32 | 4 |  |
| Pop.#5 | 5-18 | 2 | 2 | >32 | 8 |  |
| Pop.#5 | 5-19 | 2 | 0.5 | >32 | 2 |  |
| Pop.#5 | 5-2 | 2 | 0.5 | >32 | 1 |  |
| Pop.#5 | 5-20 | 2 | 0.5 | >32 | 2 |  |
| Pop.#5 | 5-21 | >16 | 4 | 32 | 2 |  |
| Pop.#5 | 5-22 | >16 | 8 | >32 | 32 | TR 34 /L98H |
| Pop.#5 | 5-23 | >16 | 2 | >32 | 8 |  |
| Pop.#5 | 5-24 | 8 | 0.25 | >32 | 16 |  |
| Pop.#5 | 5-3 | >16 | 0.5 | >32 | 2 |  |
| Pop.#5 | 5-4 | 1 | 0.5 | >32 | 2 |  |
| Pop.#5 | 5-5 | 2 | 0.5 | >32 | 32 |  |
| Pop.#5 | 5-6 | 2 | 4 | >32 | 2 |  |
| Pop.#5 | 5-7 | 2 | 0.5 | 32 | 2 |  |
| Pop.#5 | 5-8 | 1 | 1 | >32 | 2 |  |
| Pop.#5 | 5-9 | 2 | 0.5 | >32 | 2 |  |
| Pop.#6 | 6-1 | >16 | 2 | >32 | 16 |  |
| Pop.#6 | 6-10 | >16 | >16 | >32 | 32 |  |
| Pop.#6 | 6-11 | 8 | 2 | >32 | 8 |  |
| Pop.#6 | 6-12 | 16 | 1 | >32 | 8 |  |
| Pop.#6 | 6-13 | >16 | >16 | >32 | >32 |  |
| Pop.#6 | 6-14 | >16 | 16 | >32 | >32 |  |
| Pop.#6 | 6-15 | 16 | 4 | >32 | 4 |  |
| Pop.#6 | 6-16 | 16 | 2 | >32 | 8 |  |
| Pop.#6 | 6-17 | 4 | 1 | >32 | 4 |  |
| Pop.#6 | 6-18 | >16 | >16 | >32 | >32 | TR 34 /L98H |
| Pop.#6 | 6-19 | >16 | 4 | >32 | 16 |  |
| Pop.#6 | 6-2 | 4 | 4 | >32 | 8 |  |
| Pop.#6 | 6-20 | 8 | 1 | >32 | 8 |  |
| Pop.#6 | 6-21 | 8 | 1 | >32 | 4 |  |
| Pop.#6 | 6-22 | 4 | 1 | >32 | 4 |  |
| Pop.#6 | 6-23 | 4 | 2 | >32 | 2 |  |
| Pop.#6 | 6-24 | 8 | 2 | >32 | 8 |  |
| Pop.#6 | 6-25 | 4 | 2 | >32 | 4 |  |
| Pop.#6 | 6-26 | >16 | 1 | >32 | 4 |  |
| Pop.#6 | 6-27 | 4 | 1 | >32 | 8 |  |
| Pop.#6 | 6-28 | 4 | 1 | >32 | 4 |  |
| Pop.#6 | 6-3 | 4 | 16 | >32 | >32 |  |
| Pop.#6 | 6-4 | 16 | 2 | >32 | 8 |  |
| Pop.#6 | 6-5 | 8 | 2 | >32 | 8 |  |
| Pop.#6 | 6-6 | 4 | 2 | >32 | 4 |  |
| Pop.#6 | 6-7 | 4 | 1 | >32 | 4 |  |
| Pop.#6 | 6-8 | 4 | 2 | >32 | 4 |  |
| Pop.#6 | 6-9 | 4 | 4 | >32 | 8 |  |
| Pop.#7 | 7-1 | 8 | 2 | >32 | 4 |  |
| Pop.#7 | 7-10 | 8 | 1 | >32 | 8 |  |
| Pop.#7 | 7-11 | 4 | 1 | >32 | 4 |  |
| Pop.#7 | 7-12 | 4 | 4 | >32 | 32 |  |
| Pop.#7 | 7-13 | 4 | 1 | >32 | 2 |  |
| Pop.#7 | 7-14 | 4 | 1 | >32 | 2 |  |
| Pop.#7 | 7-15 | >16 | >16 | >32 | 32 | TR 34 /L98H |
| Pop.#7 | 7-16 | >16 | 2 | >32 | 8 |  |
| Pop.#7 | 7-17 | 4 | 2 | >32 | 2 |  |
| Pop.#7 | 7-18 | 4 | 0.5 | >32 | 2 |  |
| Pop.#7 | 7-19 | 8 | 2 | >32 | 8 |  |
| Pop.#7 | 7-2 | 16 | 2 | >32 | 4 |  |
| Pop.#7 | 7-20 | 16 | 1 | >32 | 2 |  |
| Pop.#7 | 7-3 | 4 | 1 | >32 | 4 |  |
| Pop.#7 | 7-4 | 2 | 2 | >32 | 8 |  |
| Pop.#7 | 7-5 | 4 | 2 | >32 | 4 |  |
| Pop.#7 | 7-6 | 8 | 4 | >32 | 4 |  |
| Pop.#7 | 7-7 | 8 | 2 | >32 | 4 |  |
| Pop.#7 | 7-8 | 4 | 1 | >32 | 2 |  |
| Pop.#7 | 7-9 | 16 | 2 | >32 | 4 |  |
| Pop.#8 | 8-1 | 4 | 2 | >32 | 4 |  |
| Pop.#8 | 8-10 | >16 | 8 | >32 | 32 | TR 34 /L98H |
| Pop.#8 | 8-11 | >16 | 4 | >32 | 32 |  |
| Pop.#8 | 8-12 | >16 | 0.5 | >32 | 32 |  |
| Pop.#8 | 8-13 | 8 | 2 | >32 | 8 |  |
| Pop.#8 | 8-14 | 8 | 0.25 | >32 | 0.5 |  |
| Pop.#8 | 8-15 | >16 | 16 | >32 | >32 | TR 34 /L98H |
| Pop.#8 | 8-16 | >16 | 8 | >32 | 32 | TR 34 /L98H |
| Pop.#8 | 8-17 | >16 | 1 | >32 | 16 |  |
| Pop.#8 | 8-18 | 8 | 0.25 | >32 | 1 |  |
| Pop.#8 | 8-19 | 8 | 0.5 | >32 | 4 |  |
| Pop.#8 | 8-2 | 4 | >16 | >32 | 16 | TR 46 /Y121F/T289A |
| Pop.#8 | 8-20 | 4 | 0.25 | >32 | 1 |  |
| Pop.#8 | 8-21 | 4 | 0.25 | >32 | 2 |  |
| Pop.#8 | 8-22 | 8 | 0.25 | >32 | 4 |  |
| Pop.#8 | 8-23 | 4 | 16 | >32 | >32 |  |
| Pop.#8 | 8-24 | 4 | 0.5 | >32 | 1 |  |
| Pop.#8 | 8-25 | 4 | 0.5 | >32 | 4 |  |
| Pop.#8 | 8-3 | >16 | 16 | >32 | 32 | TR 34 /L98H |
| Pop.#8 | 8-4 | >16 | 4 | >32 | 32 |  |
| Pop.#8 | 8-5 | 2 | 1 | >32 | 2 |  |
| Pop.#8 | 8-6 | 8 | 0.5 | >32 | 4 |  |
| Pop.#8 | 8-7 | 8 | 0.25 | >32 | 0.5 |  |
| Pop.#8 | 8-8 | 4 | 0.25 | >32 | 1 |  |
| Pop.#8 | 8-9 | >16 | 0.5 | >32 | 8 |  |
| Pop.#9 | 9-1 | >16 | 8 | >32 | >32 |  |
| Pop.#9 | 9-10 | >16 | >16 | >32 | >32 | TR 34 /L98H/S297T/F495I |
| Pop.#9 | 9-11 | >16 | 2 | >32 | 8 |  |
| Pop.#9 | 9-12 | >16 | 4 | >32 | 8 |  |
| Pop.#9 | 9-13 | >16 | 8 | >32 | 32 | TR 34 /L98H |
| Pop.#9 | 9-14 | >16 | 4 | >32 | 16 |  |
| Pop.#9 | 9-15 | >16 | 8 | >32 | >32 |  |
| Pop.#9 | 9-16 | >16 | 16 | >32 | >32 |  |
| Pop.#9 | 9-17 | >16 | >16 | >32 | >32 |  |
| Pop.#9 | 9-18 | >16 | >16 | >32 | >32 | TR 34 /L98H |
| Pop.#9 | 9-19 | >16 | 2 | >32 | 8 |  |
| Pop.#9 | 9-2 | >16 | 16 | >32 | >32 |  |
| Pop.#9 | 9-20 | >16 | 8 | >32 | 32 |  |
| Pop.#9 | 9-21 | >16 | >16 | >32 | 32 | TR 34 /L98H |
| Pop.#9 | 9-22 | >16 | 8 | >32 | 32 | TR 34 /L98H |
| Pop.#9 | 9-23 | >16 | >16 | >32 | 32 | TR 34 /L98H |
| Pop.#9 | 9-24 | >16 | >16 | >32 | >32 |  |
| Pop.#9 | 9-25 | 8 | 4 | >32 | 1 |  |
| Pop.#9 | 9-26 | >16 | >16 | >32 | 32 | TR 34 /L98H |
| Pop.#9 | 9-27 | >16 | 4 | >32 | 32 |  |
| Pop.#9 | 9-28 | >16 | 8 | >32 | 32 |  |
| Pop.#9 | 9-3 | >16 | 8 | >32 | 32 | TR 34 /L98H |
| Pop.#9 | 9-4 | 16 | >16 | >32 | >32 |  |
| Pop.#9 | 9-5 | >16 | >16 | >32 | >32 |  |
| Pop.#9 | 9-6 | >16 | >16 | >32 | >32 | TR 34 /L98H |
| Pop.#9 | 9-7 | >16 | 8 | >32 | 16 | TR 34 /L98H |
| Pop.#9 | 9-8 | >16 | >16 | >32 | >32 | TR 46 /Y121F/T289A |
| Pop.#9 | 9-9 | >16 | >16 | >32 | >32 |  |
