# Supplementary material for: Extensive Genetic Diversity and Widespread Azole Resistance in Greenhouse Populations of Aspergillus fumigatus in Yunnan, China
Source: mSphere. 2021 Feb 10;6(1):e00066-21. doi: 10.1128/mSphere.00066-21 (PMC8544883; doi:10.1128/mSphere.00066-21)
Supplement: TABLE S2 [file msphere.00066-21-st002.doc]

**Table S2-1** Summary results of AMOVA within and among populations of the *A. fumigatus* isolates from different greenhouses.

| Source | df | SS | MS | Est. Var. | % | AMOVA Statistics | Value | P |
| --- | --- | --- | --- | --- | --- | --- | --- | --- |
| Among Pops | 8 | 44.978 | 5.622 | 0.075 | 2% | PhiPT | 0.019 | 0.001 |
| Within Pops | 196 | 770.076 | 3.929 | 3.929 | 98% | PhiPT | 0.019 | 0.001 |
| Total | 204 | 815.054 |  | 4.004 | 100% |  |  |  |

**Table S2-2** Summary results of AMOVA within and among geographic populations of of *A. fumigatus* from around the world.

| Source | df | SS | MS | Est. Var. | % | AMOVA Statistics | Value | P |
| --- | --- | --- | --- | --- | --- | --- | --- | --- |
| Among Pops | 11 | 148.402 | 13.491 | 0.194 | 5% | PhiPT | 0.051 | 0.001 |
| Within Pops | 864 | 3,355.576 | 3.884 | 3.884 | 95% | PhiPT | 0.051 | 0.001 |
| Total | 875 | 3,503.978 |  | 4.078 | 100% |  |  |  |
