# Supplementary material for: Extensive Genetic Diversity and Widespread Azole Resistance in Greenhouse Populations of Aspergillus fumigatus in Yunnan, China
Source: mSphere. 2021 Feb 10;6(1):e00066-21. doi: 10.1128/mSphere.00066-21 (PMC8544883; doi:10.1128/mSphere.00066-21)
Supplement: TABLE S3 [file msphere.00066-21-st003.doc]

**Table S3** Pairwise differentiations among nine greenhouse populations of *A. fumigatus* (after clone-correction).

| **Pop.#1** | **Pop.#2** | **Pop.#3** | **Pop.#4** | **Pop.#5** | **Pop.#6** | **Pop.#7** | **Pop.#8** | **Pop.#9** |  |
| --- | --- | --- | --- | --- | --- | --- | --- | --- | --- |
|  | 0.117 | 0.466 | 0.329 | 0.016 | 0.003 | 0.419 | 0.127 | 0.056 | **Pop.#1** |
| 0.009 |  | 0.071 | 0.135 | 0.003 | 0.175 | 0.061 | 0.012 | 0.001 | **Pop.#2** |
| 0.000 | 0.016 |  | 0.153 | 0.081 | 0.054 | 0.476 | 0.174 | 0.048 | **Pop.#3** |
| 0.002 | 0.007 | 0.008 |  | 0.190 | 0.477 | 0.326 | 0.025 | 0.001 | **Pop.#4** |
| 0.022 | 0.035 | 0.019 | 0.006 |  | 0.435 | 0.013 | 0.002 | 0.001 | **Pop.#5** |
| 0.023 | 0.006 | 0.018 | 0.000 | 0.000 |  | 0.028 | 0.003 | 0.001 | **Pop.#6** |
| 0.000 | 0.014 | 0.000 | 0.003 | 0.022 | 0.017 |  | 0.218 | 0.010 | **Pop.#7** |
| 0.010 | 0.027 | 0.012 | 0.018 | 0.041 | 0.027 | 0.006 |  | 0.058 | **Pop.#8** |
| 0.016 | 0.050 | 0.022 | 0.043 | 0.059 | 0.058 | 0.031 | 0.022 |  | **Pop.#9** |

Note: PhiPT values are in the lower diagonal of the pairwise comparison table. P values based on 999 permutations are shown at top right above the diagonal
