# Supplementary material for: Extensive Genetic Diversity and Widespread Azole Resistance in Greenhouse Populations of Aspergillus fumigatus in Yunnan, China
Source: mSphere. 2021 Feb 10;6(1):e00066-21. doi: 10.1128/mSphere.00066-21 (PMC8544883; doi:10.1128/mSphere.00066-21)
Supplement: TABLE S4 [file msphere.00066-21-st004.doc]

***Table S4*** *Geographic information of strains of* *A. fumigatus from other regions in comparisons with the greenhouse populations from Yunnan, China.*

| **Geographic population** | **Country (Region)** | **Number of isolates in each country or region** | **Total number of isolates in each population** | |
| --- | --- | --- | --- | --- |
|  |  |  | **Before clone correction** | **After Clone correction** |
| **America** | Brazil | 2 | 50 | 47 |
|  | Colombia | 34 |  |  |
|  | Cuba | 14 |  |  |
| **South Asia** | India | 19 | 26 | 21 |
|  | Nepal | 7 |  |  |
| **East Asia** | China | 34 | 40 | 40 |
|  | Japan | 6 |  |  |
| **Middle Asia** | Kuwait | 2 | 14 | 13 |
|  | Iran | 12 |  |  |
| **Africa** | Tanzania | 13 | 13 | 3 |
| **South Europe** | Romania | 10 | 15 | 11 |
|  | Italy | 5 |  |  |
| **Middle Europe** | Germany | 11 | 11 | 9 |
| **North Europe** | Denmark | 14 | 14 | 10 |
| **West Europe** | France | 17 | 538 | 496 |
|  | Ireland | 19 |  |  |
|  | Netherlands | 502 |  |  |
| **Oceanica** | Australia | 16 | 16 | 14 |
| **Unclear** | Unclear | 13 | 13 | 13 |
| **Yunnan_China** | Yunnan_China | 233 | 233 | 199 |
| **Total** | | | **983** | **876** |
