# Supplementary material for: Extensive Genetic Diversity and Widespread Azole Resistance in Greenhouse Populations of Aspergillus fumigatus in Yunnan, China
Source: mSphere. 2021 Feb 10;6(1):e00066-21. doi: 10.1128/mSphere.00066-21 (PMC8544883; doi:10.1128/mSphere.00066-21)
Supplement: TABLE S5 [file msphere.00066-21-st005.doc]

**Table S5** Pairwise differentiations between regional populations of *A. fumigatus* from different parts of the world.

| America | South Asia | East Asia | Middle Asia | Africa | South Europe | Middle Europe | North Europe | West Europe | Oceania | Unclear source | Yunnan_China |  |
| --- | --- | --- | --- | --- | --- | --- | --- | --- | --- | --- | --- | --- |
|  | 0.001 | 0.001 | 0.002 | 0.001 | 0.004 | 0.038 | 0.017 | 0.001 | 0.001 | 0.002 | 0.001 | America |
| 0.057 |  | 0.134 | 0.398 | 0.037 | 0.034 | 0.003 | 0.002 | 0.001 | 0.255 | 0.002 | 0.001 | South Asia |
| 0.047 | 0.006 |  | 0.433 | 0.003 | 0.016 | 0.008 | 0.001 | 0.001 | 0.007 | 0.001 | 0.001 | East Asia |
| 0.063 | 0.001 | 0.000 |  | 0.011 | 0.123 | 0.011 | 0.001 | 0.006 | 0.008 | 0.008 | 0.001 | Middle Asia |
| 0.183 | 0.070 | 0.120 | 0.099 |  | 0.062 | 0.008 | 0.003 | 0.002 | 0.012 | 0.004 | 0.001 | Africa |
| 0.048 | 0.038 | 0.025 | 0.021 | 0.144 |  | 0.139 | 0.423 | 0.271 | 0.039 | 0.288 | 0.001 | South Europe |
| 0.032 | 0.057 | 0.033 | 0.041 | 0.187 | 0.029 |  | 0.036 | 0.050 | 0.001 | 0.013 | 0.001 | Middle Europe |
| 0.032 | 0.083 | 0.050 | 0.070 | 0.222 | 0.000 | 0.045 |  | 0.146 | 0.004 | 0.319 | 0.001 | North Europe |
| 0.031 | 0.033 | 0.024 | 0.030 | 0.128 | 0.005 | 0.021 | 0.009 |  | 0.001 | 0.130 | 0.001 | West Europe |
| 0.063 | 0.007 | 0.025 | 0.038 | 0.109 | 0.046 | 0.066 | 0.078 | 0.036 |  | 0.006 | 0.001 | Oceania |
| 0.049 | 0.062 | 0.049 | 0.048 | 0.185 | 0.008 | 0.057 | 0.006 | 0.009 | 0.072 |  | 0.001 | Unclear source |
| 0.067 | 0.054 | 0.036 | 0.053 | 0.150 | 0.069 | 0.063 | 0.084 | 0.061 | 0.067 | 0.083 |  | Yunnan_China |

Note: PhiPT Values below diagonal. Probability, P (rand >= data) based on 999 permutations is shown above diagonal.
