# Supplementary material for: Extensive Genetic Diversity and Widespread Azole Resistance in Greenhouse Populations of Aspergillus fumigatus in Yunnan, China
Source: mSphere. 2021 Feb 10;6(1):e00066-21. doi: 10.1128/mSphere.00066-21 (PMC8544883; doi:10.1128/mSphere.00066-21)

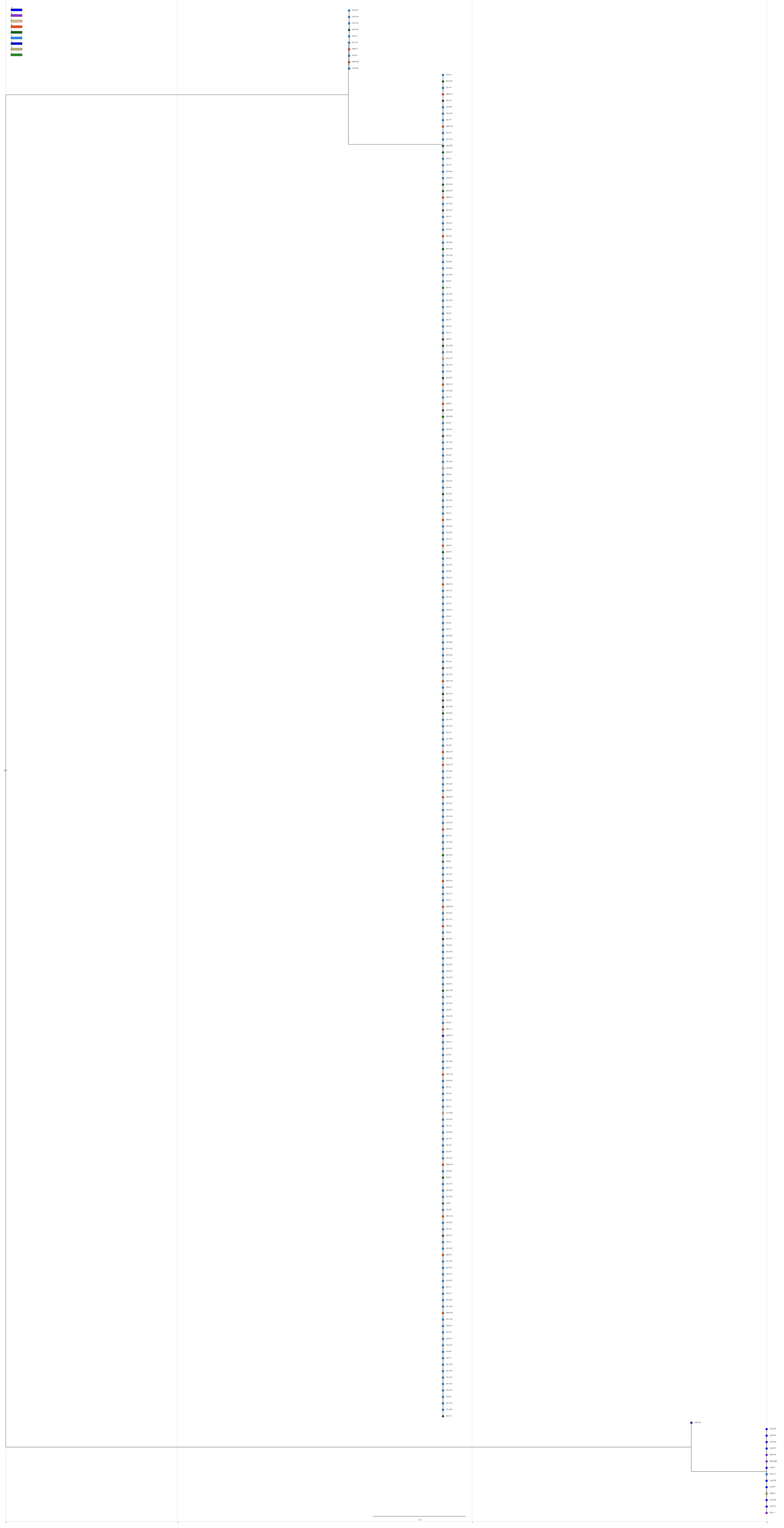

Supplement: FIG S1 [file msphere.00066-21-sf001.pdf]
