# Supplementary material for: Extensive Genetic Diversity and Widespread Azole Resistance in Greenhouse Populations of Aspergillus fumigatus in Yunnan, China
Source: mSphere. 2021 Feb 10;6(1):e00066-21. doi: 10.1128/mSphere.00066-21 (PMC8544883; doi:10.1128/mSphere.00066-21)
Supplement: FIG S3 [file msphere.00066-21-sf003.pdf]

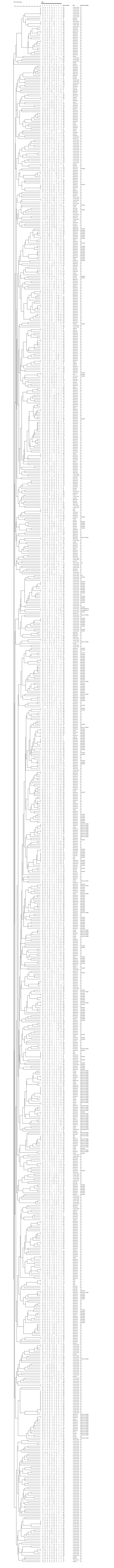
STR (<All Characters>)
STR
2A 2B 2C 3A 3B 3C 4A 4B 4C
Strain number
Pop
Promoter mutation
10 13 9 17 13 14 7 4 4 931 Yunnan_China WT
10 13 9 17 13 14 7 4 4 935 Yunnan_China WT
10 13 9 17 13 14 7 4 4 940 Yunnan_China WT
10 13 9 17 13 14 7 4 4 943 Yunnan_China WT
10 13 9 17 13 14 7 4 4 947 Yunnan_China WT
10 13 9 17 13 14 7 4 4 955 Yunnan_China WT
10 11 8 15 32 30 7 4 4 950 Yunnan_China WT
10 16 9 27 35 6 7 5 5 99 East Asia WT
11 16 9 14 24 5 7 5 5 10 Middle Asia WT
10 16 9 23 11 20 7 4 5 934 Yunnan_China WT
10 16 9 23 11 20 7 4 5 939 Yunnan_China WT
10 16 9 23 11 20 7 4 5 946 Yunnan_China WT
10 15 9 28 13 9 7 4 5 944 Yunnan_China WT
10 16 10 23 11 8 7 5 5 460 West Europ WT
10 16 10 24 11 8 7 5 5 461 West Europ WT
10 16 10 25 12 8 7 5 6 725 Middle Europ WT
10 16 10 25 12 8 7 5 6 464 West Europ WT
10 16 10 25 11 8 7 5 6 463 West Europ WT
10 16 10 22 12 12 7 5 5 314 West Europ WT
10 15 10 23 13 19 7 5 5 261 West Europ WT
10 15 10 21 12 8 7 5 5 462 West Europ WT
10 16 10 17 13 20 7 5 6 235 West Europ WT
10 16 10 17 13 20 7 5 6 236 West Europ WT
10 16 10 23 13 20 7 5 6 237 West Europ WT
10 16 10 17 13 19 7 5 6 262 West Europ WT
10 16 10 26 13 11 7 5 6 339 West Europ WT
10 14 10 17 13 14 7 5 6 300 West Europ WT
10 14 10 17 13 13 7 5 6 305 West Europ WT
10 17 10 18 11 13 7 5 6 304 West Europ WT
10 17 10 16 11 12 7 5 6 315 West Europ WT
10 15 10 18 12 12 7 5 6 316 West Europ WT
15 20 10 20 12 7 14 5 680 America WT
15 20 10 19 8 14 8 4 30 925 Yunnan_China WT
16 19 13 14 33 24 17 4 6 845 Yunnan_China WT
15 12 9 18 31 8 17 4 6 920 Yunnan_China WT
14 20 12 10 31 12 17 5 677 America WT
9 13 17 52 7 23 16 7 5 82 East Asia WT
23 16 8 59 10 21 12 10 8 220 West Europ WT
23 16 8 59 10 20 12 10 8 248 West Europ WT
23 16 8 59 11 20 12 10 8 250 West Europ WT
23 16 8 56 10 20 12 10 8 247 West Europ WT
23 16 8 56 10 13 12 10 8 307 West Europ WT
23 16 8 67 10 20 12 10 8 249 West Europ WT
23 16 8 58 10 19 12 10 8 269 West Europ WT
12 16 15 23 18 12 12 10 93 East Asia WT
18 22 10 26 13 23 12 10 5 83 East Asia WT
23 22 13 21 13 25 12 10 9 787 Yunnan_China WT
23 22 10 36 10 0 12 0 7 962 Yunnan_China WT
23 22 9 27 10 9 10 8 7 978 Yunnan_China WT
23 21 13 65 12 12 12 11 12 312 West Europ WT
23 21 8 52 15 12 16 11 8 323 West Europ WT
23 11 26 33 17 25 10 11 12 80 East Asia WT
20 16 10 29 27 16 12 15 12 50 South Asia WT
20 16 13 30 29 7 12 12 10 8 Middle Asia WT
20 16 14 8 17 23 5 11 10 43 East Asia WT
18 12 14 27 11 14 9 11 7 296 West Europ WT
18 12 14 16 9 14 22 11 8 297 West Europ WT
18 12 13 31 11 14 15 8 8 686 Oceanica WT
18 12 13 31 11 14 15 8 8 701 Oceanica WT
18 12 14 14 22 13 18 11 5 5 Middle Asia WT
18 12 18 34 23 20 9 8 8 57 America WT
17 12 18 36 12 22 19 8 8 192 West Europ WT
18 17 14 33 23 24 15 5 8 181 West Europ WT
18 17 14 33 23 23 16 5 8 188 West Europ WT
26 22 25 23 11 22 14 9 8 84 East Asia WT
25 22 20 47 11 10 19 14 8 360 West Europ WT
19 26 9 31 11 36 19 9 8 683 North Europ WT
25 16 23 22 11 20 12 11 8 222 West Europ WT
25 16 23 22 11 19 12 11 8 255 West Europ WT
25 16 23 21 11 21 12 11 8 206 West Europ WT
25 16 23 22 11 21 12 11 8 207 West Europ WT
26 16 23 22 11 20 12 11 8 223 West Europ WT
25 16 23 23 11 20 13 11 8 224 West Europ WT
25 22 19 28 11 19 15 11 8 674 America WT
20 24 22 34 11 33 12 11 8 638 America WT
20 12 15 17 11 33 7 11 8 731 South Asia WT
